# Within and beyond the stringent response-RSH and (p)ppGpp in plants

**DOI:** 10.1007/s00425-017-2780-y

**Published:** 2017-09-25

**Authors:** Justyna Boniecka, Justyna Prusińska, Grażyna B. Dąbrowska, Anna Goc

**Affiliations:** 1 Department of Genetics, Nicolaus Copernicus University in Toruń, Lwowska 1, 87-100 Toruń, Poland; 2 School of Life Sciences, University of Warwick, Coventry, CV4 7AL UK

**Keywords:** RelA/SpoT homologs, Alarmones, Plant growth and development, Stress response, Photosynthesis, Senescence

## Abstract

Plant RSH proteins are able to synthetize and/or hydrolyze unusual nucleotides called (p)ppGpp or alarmones. These molecules regulate nuclear and chloroplast transcription, chloroplast translation and plant development and stress response.

Homologs of bacterial RelA/SpoT proteins, designated RSH, and products of their activity, (p)ppGpp—guanosine tetra—and pentaphosphates, have been found in algae and higher plants. (p)ppGpp were first identified in bacteria as the effectors of the stringent response, a mechanism that orchestrates pleiotropic adaptations to nutritional deprivation and various stress conditions. (p)ppGpp accumulation in bacteria decreases transcription—with exception to genes that help to withstand or overcome current stressful situations, which are upregulated—and translation as well as DNA replication and eventually reduces metabolism and growth but promotes adaptive responses. In plants, RSH are nuclei-encoded and function in chloroplasts, where alarmones are produced and decrease transcription, translation, hormone, lipid and metabolites accumulation and affect photosynthetic efficiency and eventually plant growth and development. During senescence, alarmones coordinate nutrient remobilization and relocation from vegetative tissues into seeds. Despite the high conservancy of RSH protein domains among bacteria and plants as well as the bacterial origin of plant chloroplasts, in plants, unlike in bacteria, (p)ppGpp promote chloroplast DNA replication and division. Next, (p)ppGpp may also perform their functions in cytoplasm, where they would promote plant growth inhibition. Furthermore, (p)ppGpp accumulation also affects nuclear gene expression, i.a., decreases the level of *Arabidopsis* defense gene transcripts, and promotes plants susceptibility towards *Turnip mosaic virus*. In this review, we summarize recent findings that show the importance of RSH and (p)ppGpp in plant growth and development, and open an area of research aiming to understand the function of plant RSH in response to stress.

## Introduction

Prokaryotes have developed different types of stress responses, including chemotaxis, the SOS response and the stringent response (Dabrowska et al. 2006b). The latter was first identified in *Escherichia coli* as a reaction to amino acid deprivation (Cashel and Gallant 1969). Further experiments showed that it also takes place under limitation of other nutrients, e.g., carbon (Flardh et al. 1994; Gentry and Cashel 1996), iron (Vinella et al. 2005), fatty acid (Seyfzadeh et al. 1993; Battesti and Bouveret 2006), phosphate (Spira et al. 1995) as well as during various environmental stresses such as temperature change (Gallant et al. 1977; English et al. 2011).

Under amino acid limitation, in *E. coli*, attachment of uncharged tRNA in the ribosome acceptor site activates the ribosome-associated RelA protein to synthesize pppGpp and ppGpp nucleotides [hereafter referred to as (p)ppGpp] via the transfer of pyrophosphate from ATP to the 3′ site of GTP or GDP, respectively (reviewed in Hauryliuk et al. 2015). According to other model, RelA is rather recruited to ribosomes when uncharged tRNA is already bound in the ribosome acceptor site, then activated to produce (p)ppGpp (Wendrich et al. 2002; Brown et al. 2016), and afterwards dissociates to find another ribosome-uncharged tRNA complex (Wendrich et al. 2002). According to some researchers, RelA dissociates from the ribosome to perform several rounds of (p)ppGpp synthesis in the ribosome-unattached state (English et al. 2011). On the other hand, other scientists stated that RelA is actively displaced from ribosome during translation but not during its working times. RelA conformation required for (p)ppGpp synthesis is stabilized when the protein is bound to uncharged tRNA on ribosome (Loveland et al. 2016), undermining the aforementioned RelA “hop on-hop off” model (English et al. 2011).

(p)ppGpp act swiftly and robustly to regulate molecular targets, such as transcription (Traxler et al. 2011), translation (Milon et al. 2006; Mitkevich et al. 2010), chromosomal and various plasmid DNA replication (Levine et al. 1991; Wegrzyn 1999; Wang et al. 2007b; Maciag et al. 2010), and eventually affect growth (Potrykus and Cashel 2008) as well as bacterial virulence (Dalebroux et al. 2010). The changes invoked by alarmones are hallmarks of the stringent response, whose aim is to prevent excessive energy usage during unfavorable conditions. However, basal (p)ppGpp levels are also effective and modulate bacterial growth, perform housekeeping functions that regulate general metabolism and are even responsible for bacterial antibiotic survival (Potrykus et al. 2011; Kriel et al. 2012; Gaca et al. 2013).

In *E. coli*, (p)ppGpp are hydrolyzed by SpoT, which removes 3′ site pyrophosphate from pppGpp or ppGpp and generates GTP or GDP, respectively. SpoT is actually a bifunctional protein that is capable of (p)ppGpp degradation and synthesis (Xiao et al. 1991). Other studies show the existence of non-RelA/SpoT enzymes metabolizing (p)ppGpp (reviewed in Hauryliuk et al. 2015; Steinchen and Bange 2016) as well as of other alarmones (i.e., pGp, ppGp, pGpp). Each of them, along with pppGpp and ppGpp, probably act in a specific manner (Ooga et al. 2009; Ito et al. 2012; Mechold et al. 2013; Gaca et al. 2015).

The accumulation of (p)ppGpp results in decreased levels of ribosomal RNA (rRNA) and transfer RNA (tRNA), due to inhibition of transcription of corresponding genes, and increased expression of stress responsive genes to ensure proper cell adaptation and survival. Alarmones are required for production and function of alternative sigma factors, i.a., sigma factor S, which is responsible for bacterial entrance into a stationary phase and survival in stress conditions (Gentry et al. 1993; Kvint et al. 2000; Dabrowska et al. 2006b). The regulation of transcription occurs either via (p)ppGpp-dependent inhibition of RNA polymerase (RNAP) (Ross et al. 2013; Zuo et al. 2013) or indirectly via the regulation of GTP pool via GDP/GTP consumption during (p)ppGpp synthesis and inhibition of enzymes involved in guanosine nucleotide biosynthesis, such as guanylate kinase (Krasny and Gourse 2004; Kriel et al. 2012; Liu et al. 2015a, b).

In bacteria, (p)ppGpp also regulate translation, DNA replication, nucleotide metabolism and other targets. The indirect way to regulate translation is via the already mentioned transcriptional inhibition of rRNA and tRNA genes. Another way to inhibit translation is by competition between GTP and (p)ppGpp binding to translational guanosine triphosphatase initiation factor If2 and elongation factor Ef-G (Milon et al. 2006; Mitkevich et al. 2010). (p)ppGpp also bind to DNA primase and thus downregulate bacterial DNA replication (Wang et al. 2007b). (p)ppGpp modify other targets as well, such as guanosine triphosphatases involved in the assembly of ribosomal small and large subunits (i.e., Obg, BipA) or lysine decarboxylase LdcI to counteract low pH. All of these mechanisms are described in recent reviews (Hauryliuk et al. 2015; Steinchen and Bange 2016).

The *E. coli* RelA and SpoT (p)ppGpp metabolizing enzymes are typical for γ- and β-proteobacteria. Both RelA and SpoT carry the synthetase domain (SYNTH domain) and thus are able to produce (p)ppGpp. However, RelA is considered as the major (p)ppGpp synthetase, since SpoT shows only weak synthetic activity. Both RelA and SpoT also carry the metal-dependent hydrolysis domain (HD domain), which appears to be involved in nucleic acid metabolism and signal transduction. However, only SpoT is able to hydrolyze (p)ppGpp, since RelA lacks the highly conserved histidine and aspartate residues in the catalytic part of the HD domain (Xiao et al. 1991; Gentry and Cashel 1996; Aravind and Koonin 1998). Since the HD domain in RelA does not perform the (p)ppGpp hydrolysis function but is not entirely lost, it was proposed to be involved in maintaining the stability of the SYNTH domain, signal transduction from the C-terminal domain (CTD) and/or intermolecular interactions (Atkinson et al. 2011). The SYNTH and HD domains are localized in the N-terminal part of RSH proteins, and their activity in *Streptococcus equisimilis* was shown to be regulated by the CTD of the protein (Mechold et al. 2002).

An SMG medium that contains only one carbon amino acid invokes metabolic imbalance and results in isoleucine starvation that in wild-type (WT) strains promotes (p)ppGpp production what helps to overcome the amino acid shortage. Since in *E. coli*, RelA is the major (p)ppGpp synthase, its mutation (*relA* ^−^) prevents alarmones production and subsequently cell growth on SMG. *spoT* ^−^ mutant is not able to survive on SMG because the unabated accumulation of (p)ppGpp is detrimental for cells. *relA* ^−^ *spoT* ^−^ double mutant behaves similarly to *relA* ^−^, but an introduction of a *RSH* transgene with only (p)ppGpp synthetic activity results in a SpoT^−^ phenotype (Uzan and Danchin 1976; Xiao et al. 1991; Cashel et al. 1996). These strains are commonly used in complementation studies to assess whether a studied RSH protein has (p)ppGpp synthetic and/or hydrolytic activity.

RelA and SpoT belong to the so-called “long” multidomain group of RSH proteins, which contain the SYNTH and the HD domains in their N-terminal part, and optionally also TGS, helical, conserved cysteine and ACT domains in their CTD part. The CTD of RSH was proposed to regulate the catalytic activity of RSH proteins and to mediate both inter- and intra-molecular interactions. However, no exact residues that accounts for these interactions were identified (Atkinson et al. 2011). Originally, the TGS sequence was found in threonyl-tRNA synthetase, guanosine triphosphatase as well as in SpoT proteins, and, based on that, the domain was proposed to have a regulatory role in ligand (most likely nucleotide) binding (Wolf et al. 1999). The ACT domain (aspartate kinase chorismate mutase TyrA) is a ligand-binding domain that is present in the proteins involved in amino acid and purine biosynthesis (Chipman and Shaanan 2001), suggesting its role in amino acid binding. Thus, both TGS and ACT domains seem to play a role in ligand binding. Recently, ACT domain was described to be actually an RNA recognition motif (RRM) domain that along with TGS domain play a role in the *E. coli* RelA interaction with the uncharged tRNA in the ribosome acceptor site (Brown et al. 2016; Loveland et al. 2016). In silico RelA and SpoT analysis showed that the TGS and ACT domains are the hotspots for rate variation, what suggests their involvement in organism adaptation towards different kinds of stresses (Atkinson et al. 2011). Indeed, the TGS domain of *E. coli* SpoT protein interacts with acyl carrier protein, the central cofactor in fatty acid synthesis, which signals fatty acids starvation to SpoT (Battesti and Bouveret 2006).

Bacteria and animals also have a single domain RSH called “short RSH” that contain either the SYNTH domain, e.g., firmicute bacteria *Streptococcus mutans* (Lemos et al. 2007), *Bacillus subtilis* (Nanamiya et al. 2008), *Vibrio cholera* (Das et al. 2009; Pal et al. 2011), or the HD domain (i.e., bacteria, metazoan) and are called Small Alarmone Synthetases (SAS) or Small Alarmone Hydrolases (SAH), respectively (Atkinson et al. 2011). One of such SAH animal examples is *Drosophila melanogaster*, *Caenorhabditis elegans* and human metazoan SpoT homologue 1 (MESH1). In bacteria, SAS and SAH accompany the long RSH and were proposed to fine-tune bacterial sensitivity and to bolster responses to the stringent response-inducing stimuli (Sun et al. 2010; Atkinson et al. 2011).

Genes encoding for (p)ppGpp synthetases and hydrolases are widespread and highly conserved in green algae (Kasai et al. 2002), a moss *Physcomitrella patens* (Sato et al. 2015), a monocotyledon plant *Oryza sativa* (Xiong et al. 2001; Tozawa et al. 2007) and dicotyledon plants, i.e., *Arabidopsis thaliana* (van der Biezen et al. 2000; Tozawa et al. 2007; Masuda et al. 2008a), *Nicotiana tabacum* (Givens et al. 2004), *Suaeda japonica* (Yamada et al. 2003), *Capsicum annnum* (Kim et al. 2009) and *Pharbitis nil* (Dabrowska et al. 2006a). The origin and evolution of the stringent response genes in plants are described in the recent review (Ito et al. 2017), according to which *RSH* genes were introduced into proto-plant cell by lateral gene transfer events from different bacterial phyla.

The aim of the review is to summarize the current state of our knowledge of RSH and (p)ppGpp in plants. We focus on RelA/SpoT Homologs (RSH) across multiple plant species, their domain structure and function, including (p)ppGpp synthetic and hydrolytic activity, gene expression, function in chloroplasts and cytosol as well as their importance for plant growth, development and abiotic and biotic stress responses.

## Distribution, structure and enzymatic activity of plant RSH proteins

Plant RSH were divided into four subgroups (RSH1–4), where RSH2 and RSH3 (due to their high similarity) were eventually put into one subgroup. It seems that the three plant RSH families (RSH1, RSH2/3 and RSH4) diverged after the separation of algae and mosses but before the separation of mosses and seed plants. The green algae *Chlamydomonas reinhardtii* has a single RSH protein, with both (p)ppGpp synthetase and hydrolase activities (Kasai et al. 2002), that does not cluster within any other plant RSH (Masuda 2012). *P. patens*, a moss which belongs to the bryophyte group, intermediates between algae and vascular plants, encodes putatively for nine RSH proteins, which according to in silico studies cluster within RSH1–4 subgroups (except for *Pp*RSH4) (Sato et al. 2015). Thus, perhaps the three RSH subgroups were established when plant species adapted to terrestrial growth (Masuda 2012).

The relatively high number of diverse *RSH* in plants is likely due to gene duplication, followed by domain loss in some subgroups and gain of the plant specific calcium-binding EF-hand motifs in the RSH4 subgroup (Atkinson et al. 2011).

It seems that bacteria and plants have developed different ways of achieving the accuracy and magnitude of RSH-mediated responses. Plants, contrary to bacteria, carry neither SAS nor SAH but only the long RSH. However, plant RSH display more complex domain combination in comparison to non-plant long RSH. Thus, in plants, the complexity of domain composition is probably the source of superior resilience and adaptation to various adversities, whereas bacteria instead equip themselves with SAS and SAH, which accompany the classical long RSH (i.a., RelA and SpoT). Nevertheless, from the evolutionary point of view, it is very intriguing that the domain structure of RSH proteins is so highly conserved across kingdoms. Both bacteria and plants long RSH carry highly similar SYNTH and HD domains. Moreover, the subfunctionalizations observed in bacteria such as loss of a functional HD domain in the RelA protein and the weak synthetic activity of SpoT also take place among plant RSH (Atkinson et al. 2011).

**Fig. 1:**
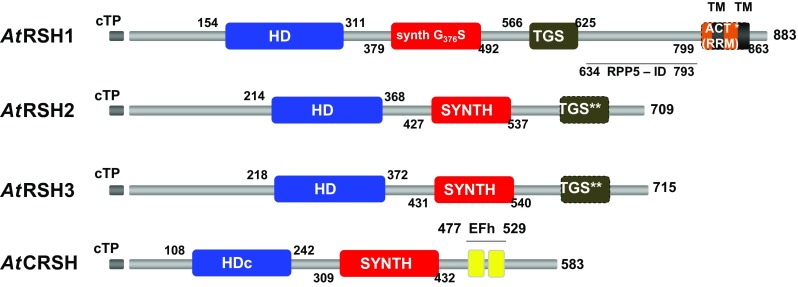
Schematic representation of the conserved domains structure in *Arabidopsis thaliana* RSH proteins. cTP, chloroplast target peptide (gray); HD, (p)ppGpp hydrolase domain (blue); SYNTH, (p)ppGpp synthetase domain; TGS, TGS regulatory domain; ACT (RRM), ACT regulatory domain (RNA recognition motif domain; orange); EFh, calcium binding EF hand motifs constituting on EF hand domain (yellow); synth G_376_S, glycine into serine substitution at position 376 AA in the SYNTH domain that abolishes (p)ppGpp synthetase activity; HDc, degraded HD domain in CRSH, which may be nonfunctional (Atkinson et al. 2011); TM, putative transmembrane region (811–827 and 848–864 AA; black) and RPP5-ID, RPP5 interacting domain (634–793 AA) proposed by van der Biezen et al. (2000). The figure does not include the helical domain, which was found between TGS and ACT (RRM) domains, of RSH1 (Atkinson et al. 2011). Conserved domains localization is described based on the NCBI Conserved Domains database, except TGS domain in RSH2 and RSH3 proteins, which was found by Atkinson et al. (2011). According to Ito et al. (2017), ACT (RRM) (asterisk) and TGS (double asterisk) domains are not conserved in *Arabidopsis* RSH1 and RSH2/RSH3, respectively

Among the different types of plant *RSH*, *RSH1* has the widest distribution, as it is already present in archaeplastida and chromalveolates (Atkinson et al. 2011). In recent studies with five plant phyla, *RSH1* was found among Embryophyta, Charophyta, Chlorophyta and Rhodophyta, but not in Glaucophyta (Ito et al. 2017). In most plants, RSH1 protein carries all four major domains (HD, SYNTH, TGS and ACT; Fig. 1) present in the bacterial long RSH (Atkinson et al. 2011). However, the ACT domain, described recently as an RNA recognition motif (RRM) domain (Brown et al. 2016), was shown as not conserved in *Arabidopsis* or *Physcomitrella* (asterisk in Fig. 1; Ito et al. 2017). Plant RSH1 TGS domain was proposed to play a role in RSH1–ribosome interaction in chloroplasts, similarly to its function in bacteria (Brown et al. 2016; Loveland et al. 2016; Ito et al. 2017). Even though RSH1 has the SYNTH domain, it rather functions as a (p)ppGpp hydrolase since the conserved glycine residue necessary for (p)ppGpp synthetase activity is substituted with serine (Wendrich and Marahiel 1997; Masuda et al. 2008a; Fig. 1). Although van der Biezen et al. (2000) were able to show that *Arabidopsis* RSH1 (*At*RSH1) confers phenotypes that are characteristic of ppGpp synthetase in *E. coli* and a Gram-positive bacterium *Streptomyces coelicolor*, other genetic studies show that *At*RSH1 is only a (p)ppGpp hydrolase. For example, *Arabidopsis RSH1* knock out (*rsh1*), and *RSH1*-overexpressing lines (*RSH1oe*) produce more and less (p)ppGpp, respectively, in comparison to WT plants. Additionally, *AtRSH1* expression in a slow growing *E. coli* strain that overaccumulates ppGpp significantly accelerates growth of the bacteria, whereas the expression of its allele with mutation in the HD domain maintains the bacterial slow growth phenotype (Sugliani et al. 2016). Complementation studies of *E. coli relA* ^−^ and *relA* ^−^ *spoT* ^−^ mutants with *AtRSH1* also show that *At*RSH1 protein has rather no (p)ppGpp synthetase activity (Mizusawa et al. 2008).

*RSH2* and *RSH3* are characteristic only for green plants. In *Arabidopsis*, *RSH2* and *RSH3* are the results of *RSH2* duplication, and a true *RSH3* is missing from these plants. Nevertheless, it was found in other multicellular plants; thus, it appears to be lost in the lineage to *Arabidopsis*. Atkinson et al. (2011) proposed that *At*RSH3, similarly to plant RSH2, carries the TGS domain, while other plant RSH3 have only a fragment of the TGS domain at the very end of CTD. According to recent data no TGS domain is present in *Arabidopsis* RSH2 and RSH3 proteins (Ito et al. 2017). Neither RSH2 nor RSH3 carry the ACT (RRM) domain (Fig. 1). The conserved residues required for activity of the SYNTH and HD domains are present in both RSH2 and RSH3, implying their metabolic bifunctionality (Atkinson et al. 2011). *E. coli relA* ^−^ and *relA* ^−^ *spoT* ^−^ mutants’ complementation analysis show that *At*RSH2 and *At*RSH3 display (p)ppGpp synthetic activity, which for RSH2 seems to be very strong, as *relA* ^−^ *spoT* ^−^ mutant complementation with the protein is lethal (Mizusawa et al. 2008; Sugliani et al. 2016). Overexpression of *AtRSH3* in *Arabidopsis* WT as well as double knockout *rsh2 rsh3* lines suggests that RSH3 has a (p)ppGpp synthetic activity, since both lines produce more alarmones than WT plants (Maekawa et al. 2015; Sugliani et al. 2016). However, surprisingly, the overexpression of *AtRSH2* in the *rsh2 rsh3* background did not affect (p)ppGpp level in comparison to WT (Maekawa et al. 2015). Nevertheless, *Arabidopsis* RSH2 and RSH3 function rather as synthetases, since their expression in a slow growing *E. coli* strain that overaccumulates ppGpp was not possible, likely due to (p)ppGpp overproduction (Sugliani et al. 2016). *N.* *tabacum* RSH2 (*Nt*RSH2), which shows high homology to *Arabidopsis* RSH2 and RSH3, exhibits (p)ppGpp synthetase activity based on biochemical and genetic studies. The protein complements *E. coli relA* ^−^ mutant and, similarly to *At*RSH2, is toxic to the *relA* ^−^ *spoT* ^−^ mutant, which underscores its strong (p)ppGpp synthetic activity (Givens et al. 2004). Another member of the RSH2 group, *S. japonica* RSH (*Sj*RSH), also complements *relA* ^−^ in *E. coli* what is indicative of its (p)ppGpp synthetase activity (Yamada et al. 2003). Based on biochemical and the *E. coli relA* ^−^ complementation studies, *P. patens* RSH2a and RSH2b, which cluster within the RSH2/RSH3 subgroup, are (p)ppGpp synthetases. Surprisingly, no hydrolytic activity of these proteins was shown, despite the presence of the highly conserved catalytic residues in their HD domains (Sato et al. 2015), whereas transformation of a slow growing *E. coli* strain that overaccumulates ppGpp either with *AtRSH2* or *AtRSH3* was not successful, which is probably due to (p)ppGpp overproduction (Sugliani et al. 2016).

RSH4 or Ca^2+^-activated RSH (CRSH) is the plant RSH closest to RSH1 (Atkinson et al. 2011) and appears to be present only in land plants. It carries neither the TGS nor ACT (RRM) domain. Instead, in its CTD, there are two Ca^2+^-binding EF hand motifs (Fig. 1) that are important for *A. thaliana* CRSH and *O. sativa* CRSH1 (p)ppGpp synthetic activities (Tozawa et al. 2007; Masuda et al. 2008a). Since cytosolic calcium levels change under developmental and stress-induced signals, EF hand motif-carrying proteins are able to transmit information about such stimuli (Day et al. 2002). Thus, CRSH was proposed to mediate the stringent response induced by such cues, as it is able to sense and respond to calcium fluctuations by producing (p)ppGpp. In *At*CRSH, the HD domain is degraded (Fig. 1) and probably not functional (Atkinson et al. 2011). However, *E. coli relA* ^−^ *spoT* ^−^complementation studies with *AtCRSH* did not result in bacterial cell death, which would be expected to happen if it produces (p)ppGpp but is not able to hydrolyze them (Masuda et al. 2008a). A possible explanation is that CRSH is a weak (p)ppGpp synthetase that does not cause toxic accumulation of alarmones. That would also explain why *AtCRSH* expressed in *E. coli relA* ^−^ mutant is not able to restore the WT phenotype (Mizusawa et al. 2008). It probably produces (p)ppGpp to the extent that can be regulated by SpoT. Similar complementation results as well as no hydrolase activity were shown with *Os*CRSH1 (Tozawa et al. 2007). The notion that the CRSH protein lacks (p)ppGpp hydrolytic activity is supported with studies showing no effect of the *AtCRSH* expression on the amplification of a slow growing *E. coli* strain that overaccumulates ppGpp (Sugliani et al. 2016).

Based on plant RSH in silico analysis, genetic and biochemical studies, it can be concluded that RSH1 and CRSH have only (p)ppGpp hydrolase and synthetase activity, respectively, whereas RSH2 and RSH3 have both activities (Sugliani et al. 2016).

## Role of RSH proteins and alarmones in chloroplasts

### RSH localization and function in chloroplasts

Experiments performed in 1974 implied that chloroplasts are the loci of ppGpp production, as ppGpp synthesis was shown to be mediated by *C. reinhardtii* ribosomes of chloroplastic but not cytoplasmic origin (Sy et al. 1974).

Numerous studies have clearly shown that plant RSH proteins predominantly localize in chloroplasts, which are the site of the RSH action. *C. reinhardtii* RSH translocates to chloroplasts in vitro, which depends on the plastid targeting sequence, light and ATP (Lawrence and Kindle 1997; Kasai et al. 2002). *N. tabacum* RSH2 co-purifies with chloroplasts in subcellular fractionation experiments (Givens et al. 2004). *A. thaliana* RSH2– and RSH3–GFP fusion proteins expressed in *Nicotiana benthamiana* localize to chloroplasts (Maekawa et al. 2015). Similarly, *Pp*RSH2a– and *Pp*RSH2b–GFP fusion proteins localize to chloroplasts, despite the predicted lack of the N-terminal transit peptide (Sato et al. 2015). *Arabidopsis* CRSH–GFP fusion protein expressed under control of the constitutive CaMV 35S promoter shows chloroplastic localization, which was confirmed also in a subcellular fractionation experiment followed by Western blot analysis by use of anti-CRSH antibody (Masuda et al. 2008a). Rice *Os*CRSH1 recombinant protein is imported into chloroplasts in vitro (Tozawa et al. 2007). Since *At*RSH1 was proposed to carry a RPP5 (cytoplasmic protein involved in the plant effector-triggered immunity)-interacting region and a transmembrane domain in the CTD part of the protein (Fig. 1), it was described as cytoplasmic protein that anchors in the cell membrane (van der Biezen et al. 2000). However, *At*RSH1–GFP fusion protein localizes in chloroplasts (Chen et al. 2014), and the GFP fusion protein containing *At*RSH1 putative chloroplast transit peptide translocates to chloroplasts (Mizusawa et al. 2008), implying *At*RSH1 plastid thylakoid and/or envelope membrane localization. RSH protein localization studies additionally show that RSH from each subgroup may act in a different spatio-temporal manner. As an example, *At*CRSH and *Nt*RSH2 proteins were found in chloroplast soluble and insoluble fractions, respectively (Givens et al. 2004; Masuda et al. 2008a). All these results are in agreement with around 13-fold higher levels of ppGpp in pea chloroplasts than in pea shoots (Takahashi et al. 2004).

Since SpoT-like protein from *Rhodobacter capsulatus*, bacteria that can obtain energy through photosynthesis, promotes the synthesis of photopigments, it was proposed that the stringent response regulates photosynthesis (Masuda and Bauer 2004; Masuda 2012). Because chloroplasts are descendants of photosynthetic bacteria, the (p)ppGpp-mediated regulation of photosynthesis in such prokaryotes was an argument for similar regulation of plant photosynthesis (Gray 1993; Givens et al. 2004).

In the last couple of years, progress has been made in deciphering bacterial-like stringent response in plants. It has been shown that the response regulates chloroplast processes such as transcription, translation, and production of nucleotides, hormones, lipids and metabolites. Moreover, recent findings also shed light on unique features of plant stringent response.

### (p)ppGpp-mediated regulation of transcription in chloroplasts

Chloroplast proteins are encoded by plastid (i.a., *rbcL—*large subunit of Rubisco) and nuclear genes (i.a., *RSH* and Rubisco small subunit). The chloroplast-encoded genes are transcribed by plastid-encoded RNA polymerase (PEP), which is similar to the bacterial RNAP, or nuclear-encoded plastid RNA polymerase (NEP), which is a T7 phage-like RNA polymerase (Swiatecka-Hagenbruch et al. 2007; Liere et al. 2011).

In plants, (p)ppGpp regulate transcription hypothetically in two different ways, which are similar to the ones observed in bacteria. First one resembles the allosterical regulation of *E. coli* RNAP activity that results from the direct (p)ppGpp interaction with RNAP ω and β′ subunits (Ross et al. 2013). In chloroplasts, ppGpp was shown to bind to the β’ subunit of the bacterial-like PEP to inhibit transcription in a dose-dependent manner (Sato et al. 2009). However, in plants a homolog of *E. coli* ω subunit does not exist and the β′ subunit is different in many aspects from the bacterial counterpart. Moreover, the Rpo core enzyme (α2, β, β′, β′′) of PEP is surrounded by additional nuclear-encoded subunits (the PEP-associated proteins-PAPs) that have no bacterial homologs, are essential for PEP activity in chloroplasts (Borner et al. 2015; Pfannschmidt et al. 2015) and likely prevent the direct (p)ppGpp–PEP interaction. Therefore, a direct regulation of transcription from (p)ppGpp by allosteric interaction with the polymerase appears unlikely, although it is not impossible. In addition, the interaction between (p)ppGpp and the β′ subunit was shown in vitro only (Sato et al. 2009). Thus, it remains to be demonstrated that it can also occur in vivo.

Another possible way of transcriptional regulation is the limitation of GTP pool due to its usage for alarmone production. In plastids, similarly to *B. subtilis,* the transcription of rRNA genes starts from GTP (Suzuki et al. 2003; Krasny and Gourse 2004; Swiatecka-Hagenbruch et al. 2007), suggesting that the concentration of the initiator nucleotide is crucial for their transcription and that (p)ppGpp impacts the process. (p)ppGpp also regulates GTP biosynthesis in chloroplasts as plant (rice, pea and *Arabidopsis*) guanylate kinases (GKs), catalyzing the conversion of GMP to GDP, are sensitive to (p)ppGpp in vitro (Nomura et al. 2014a). Interestingly, GK of *B. subtilis,* but not of *E. coli,* is also inhibited by alarmones (Kriel et al. 2012; Nomura et al. 2014a), showing that the targets of (p)ppGpp differ between these bacteria and further suggesting that in plants during the stringent response transcription is regulated rather in the indirect way proposed for *B. subtilis* (Krasny and Gourse 2004). Nevertheless, the inhibition of GKs by alarmones must also bring a negative feedback loop to reduce (p)ppGpp production due to substrate (GDP) exhaustion. ppGpp also regulates the activity of enzymes implicated in ATP biosynthesis, namely, adenylosuccinate synthetases (ASs). ppGpp inhibits rice *Os*AS1 and *Os*AS2 in a guanine nucleotide concentration-dependent manner (Nomura et al. 2014b). Since ATP is needed for alarmone production, the (p)ppGpp-mediated AS inhibition may serve as a negative feedback loop. Another fact that demonstrates the indirect way of the (p)ppGpp-mediated regulation of transcription in plants is the lack of DnaK suppressor (DksA)-like gene in *Arabidopsis*, which encodes RNAP-associated protein required for full and direct (p)ppGpp-mediated regulation of transcription in *E. coli* (Paul et al. 2004, 2005). Furthermore, the concentration of (p)ppGpp in chloroplasts, estimated with high-sensitive ppGpp quantification method, is ~ 3 µM (Ihara et al. 2015). Hence, taking into account that the ppGpp concentration required for 50% GK inhibition (IC50) is ~ 10 µM (Nomura et al. 2014a), Ihara et al. (2015) suggested that GK could be regulated by ppGpp in chloroplasts. In contrary, PEP, according to in vitro analysis, requires higher ppGpp concentrations (200–1000 µM; Sato et al. 2009) than found in chloroplasts. Thus, ppGpp concentration in chloroplasts seems to be sufficient to inhibit GK but not PEP (Sugliani et al. 2016). Although it is not fully understood how the transcription is regulated during the stringent response in plants, some downstream targets have been identified.

Among chloroplast genes, whose expression are reduced under (p)ppGpp accumulation, are genes transcribed mainly by PEP and encoding for the components of photosystem I (i.e., PsaB, PsaC), photosystem II (PSII; i.e., PsbA, PsbD, PsbK), translation machinery (i.e.,16S, 23S, ribosomal protein Rps14, TRNR–arginine tRNA) and RbcL. NEP transcribed genes may also be affected, for example, the ones encoding for the components of translation machinery (i.e., ribosomal protein Rps18), PEP (i.e., RNA polymerase alpha and beta subunits RpoA and RpoB) and for caseinolytic protease P1 (ClpP1), a subunit of the translocon on the inner envelope of chloroplasts (Ycf1), an ATPase of unknown function (Ycf2) as well as acetyl-CoA carboxylase beta subunit (AccD). However, the (p)ppGpp-mediated regulation of NEP-dependent genes expression is questioned as the observed changes might be due to posttranscriptional modifications, which would lead to differential regulation of turnover for PEP- and NEP-dependent transcripts (Sugliani et al. 2016). Indeed, using a method that eliminates the influence of transcript degradation, Sugliani et al. (2016) showed no major (p)ppGpp influence on the expression of NEP-dependent genes. The data obtained with the *Arabidopsis* line overexpressing *RSH3* in *rsh2 rsh3* background and accumulating higher levels of (p)ppGpp than WT plants also demonstrates reduction in the expression of both PEP- and NEP-dependent genes, i.e., *psbA*, *psbD*, *rbcL* and *accD*, *rpoA*, *clpP1*, respectively. However, the (p)ppGpp-mediated regulation of NEP-dependent genes expression might have occurred also due to differential PEP- and NEP-dependent transcripts turnover (Maekawa et al. 2015). Although the (p)ppGpp-mediated regulation of NEP-dependent genes expression is not clear, the decreased expression of chloroplast rRNA and tRNA is implicit (Sugliani et al. 2016; Abdelkefi et al. 2017).

The (p)ppGpp-mediated expression of chloroplast genes highly overlaps with their expression invoked with abscisic acid (ABA). Treatment of *Arabidopsis* plants with ABA results in significantly reduced expression of chloroplast genes encoding for PSII components, i.e., PsbE, PsbH, PsbI, PsbJ,L,E, PsbK, PsbM, PsbN-oligo, PsbZ, both in younger and older plants as well as for PsbB in older plants. It also affects the expression of other chloroplast genes, such as the ones encoding for PEP subunits (i.e., RpoA, RpoB, RpoC1, RpoC2) and for proteins of large ribosomal subunit (i.e., Rpl14, Rpl16, Rpl2, Rpl20, Rpl23, Rpl32, Rpl33) (Yamburenko et al. 2015). The decreased expression of NEP-dependent genes (i.a., *rpoB*) again shows that (p)ppGpp might also regulate NEP activity, which would contradict the earlier report (Sato et al. 2009). Another explanation for the changes in NEP-dependent transcript levels in ABA-treated samples is the reduced expression of the NEP-encoding gene as well as the high possibility that the treatment with ABA affects not only (p)ppGpp-resembling responses but also the non (p)ppGpp-invoked ones. The last hypothesis is supported with differential expression of the PSII component encoding gene *psbA*, whose transcripts are not affected under ABA treatment but are downregulated in the (p)ppGpp-accumulating lines (Maekawa et al. 2015; Yamburenko et al. 2015; Sugliani et al. 2016; Abdelkefi et al. 2017). Since ABA promotes the expression of *RSH2* and *RSH3*, it was proposed that the transcriptional de-repression invoked with ABA is mediated via the RSH-(p)ppGpp module. This idea is supported with the observation that *Arabidopsis rsh2* and *rsh3* mutants treated with ABA express even around 60% higher amount of chloroplast transcripts in comparison to WT plants (Yamburenko et al. 2015). The very high overlap of genes affected in lines overproducing (p)ppGpp and in plants treated with ABA shows that ABA-invoked effects are to some extent (p)ppGpp-mediated. The correlation of ABA responses with (p)ppGpp action is corroborated with the ABA-promoted expression of nuclear-encoded sigma factor 5 (*SIG5*), which strongly resembles the stringent response-mediated expression of alternative sigma factors, which mediate transcription of stress responsive genes in bacteria (Yamburenko et al. 2015).

### RSH/(p)ppGpp-mediated regulation of translation in chloroplasts

(p)ppGpp accumulation affects protein level in chloroplasts, leading to a decreased total amount of proteins in *Arabidopsis* plants (Maekawa et al. 2015; Sugliani et al. 2016). This is not surprising, since plastid protein synthesis system retains prokaryotic components such as 70S ribosome and translation factors involved in (p)ppGpp-mediated regulation of translation in bacteria. Enzymatic function of the pea chloroplast (p)ppGpp synthetase is associated with 70S ribosomes and sensitive to tetracycline, which also inhibits peptide synthesis in pea chloroplasts (Kasai et al. 2004). In bacteria, ppGpp directly inhibits translation through interaction with factors involved in translation initiation and elongation, If2 and Ef-G, respectively (Milon et al. 2006; Mitkevich et al. 2010). Since plants have their chloroplastic homologs (Akkaya and Breitenberger 1992; Miura et al. 2007; Nomura et al. 2012), pea chloroplast EF-G is active on *E. coli* ribosomes (Akkaya and Breitenberger 1992) and ppGpp inhibits peptide elongation in a chloroplast translation system in vitro (Nomura et al. 2012), it is highly possible that (p)ppGpp regulate translation via the inhibition of these proteins in chloroplasts. Since translation is the major consumer of ATP and GTP nucleotides for aminoacyl–tRNA synthesis and elongation factor recycling and the level of these nucleotides is regulated by (p)ppGpp, the indirect involvement of (p)ppGpp in the regulation of translation is also possible (Nomura et al. 2012). Furthermore, *At*RSH1 interacts with a chloroplastic protein from the Obg family, whose members play a role in ribosome assembly and thus may have an impact on translation (Chen et al. 2014).

(p)ppGpp accumulation in chloroplasts controls the production of chloroplast-encoded proteins because the overexpression of *Arabidopsis RSH3*, which accumulates high amounts of (p)ppGpp, causes a strong reduction in the level of chloroplast-encoded PsbA, the subunit of the reaction center from PSII (RCII). However, it does not affect the production of nucleus-encoded PSII light-harvesting complexes (LHCII) and thus decreases the RCII/LHCII ratio. Since LHCII are rich in chlorophyll b and highly fluorescent, the most probable explanation of strong basal chlorophyll fluorescence and the reduction in the maximal efficiency of PSII, which is annotated for both *RSH2* and *RSH3* overexpressing lines, is that low RCII/LHCII causes stoichiometric displacement of LHCII from RCII fraction (Maekawa et al. 2015; Sugliani et al. 2016). Concomitantly, *Arabidopsis rsh2*, *rsh3*, *crsh* and *rsh2 rsh3* mutants exhibit significantly weaker basal chlorophyll fluorescence than WT plants, which is even weaker in the quadruple mutant of all four *Arabidopsis* RSH. Similarly, the RSH1 overproducing line shows weaker basal chlorophyll fluorescence, whereas *rsh1* displays the opposite phenotype due to high (p)ppGpp accumulation (Sugliani et al. 2016). However, in another study, no changes in parameters indicative of the regulation of photosynthetic light reactions in *rsh2 rsh3* mutant were shown (Maekawa et al. 2015).

In addition to PsbA protein downregulation, overexpression of *Arabidopsis RSH3* negatively affects the levels of chloroplast- and nuclear-encoded PsaB, RbcL (PEP-dependent), chloroplast f1 (Cf_1_)-β (NEP-dependent) and Rubisco small subunit proteins, respectively. Surprisingly, despite decreased transcript levels, AccD protein level increases in line accumulating alarmones (Maekawa et al. 2015; Sugliani et al. 2016).

(p)ppGpp is not able to inhibit translation as fast as translation inhibitor lincomycin, which is likely due to high (p)ppGpp concentration requirement for that purpose. While the lincomycin induced significant inhibition of translation occurs 24 h after the expression of a constitutive (p)ppGpp synthetase domain from *E. coli* in *Arabidopsis*, for (p)ppGpp it takes 72 h (Sugliani et al. 2016). Thus, the low production of chloroplast proteins invoked with (p)ppGpp is the result of the low level of components that constitute the chloroplastic translational machinery and transcripts undergoing translation rather than a direct effect on translation.

### (p)ppGpp regulate the level of hormones, lipids and metabolites in chloroplasts

(p)ppGpp regulate the level of salicylic acid (SA), a hormone that the main biosynthetic pathway takes place in chloroplasts. The accumulation of alarmones in the *Arabidopsis RSH3oe* plants causes reduction of the hormone level, whereas the decreased amount of (p)ppGpp in the *Arabidopsis* quadruple mutant line (*RSH1*–*RSH3* knock out and *CRSH* knock down) correlates with increased SA level (Sugliani et al. 2016; Abdelkefi et al. 2017).

Lipid production occurs within chloroplasts, and (p)ppGpp regulate their levels. *AtRSH3* overexpression in *rsh2 rsh3* background leads to lower content of all molecular species of fatty acid (significantly of 16:0, 16:1, 16:2, 16:3, 18:1, 18:2, 18:3) but has no major impact on their composition. Significant downregulation was annotated for the polar glycerolipids such as monogalactosyldiacylglycerol (MGDG) and sulphoquinovosyldiacylglycerol, whereas phosphatidylethanolamine and phosphatidylcholine were shown to be more abundant. Similarly, the levels of most of the tested metabolites (e.g., Krebs cycle metabolites, fumarate, malate, gluthamine synthetase/glutamine oxoglutarate, glutamate, many amino acids) were lower in comparison to WT plants (Maekawa et al. 2015).

AccD is an important subunit of the ACC complex. The function of the complex is to regulate the metabolism of fatty acids. When the enzyme is active, the product, malonyl-CoA, a building block for new fatty acids, is produced. The precursor of MGDG is linolenic acid, a 18:3 fatty acid. While the level of MGDG is downregulated in the line overproducing (p)ppGpp, the AccD protein accumulation is enhanced. On the first glance this appears to be a contradiction, however, it is unknown how the complete ACC complex behaves. In addition, *accD* appears to be special among plastid genes as it is probably the only plastid protein-coding gene that transcript and protein levels are constitutively high during tomato fruit ripening (Kahlau and Bock 2008).

(p)ppGpp were also shown to promote tolerance to nutrient starvation as in nitrogen-limited media the *Arabidopsis* line overproducing (p)ppGpp (the *AtRSH3oe* in *rsh2 rsh3* background) stays green and does not accumulate starch, glucose, sucrose and Rubisco, the source of nitrogen during nitrogen starvation, to the levels observed in WT and *rsh2 rsh3* plants, which is generally a form of maintaining the nitrogen–carbon balance. Indeed, the carbon/nitrogen ratio in *AtRSH3oe* plants under nitrogen deficiency is significantly lower than in WT plants, suggesting higher nitrogen deficiency tolerance of the mutant plants (Honoki et al. 2017). Similarly, in phosphorous-limited conditions, the same line accumulates significantly lower levels of anthocyanins, which is again indicative either of higher nutrient deprivation tolerance or the lack of starvation sensing (Maekawa et al. 2015). Nitrogen deficiency (p)ppGpp-mediated tolerance occurs also likely due to reduced chlorophyll content, changes in photosynthetic parameters as well as pronounced changes in metabolite levels in *AtRSH3oe* plants in comparison to WT plants. Thus, it was proposed that (p)ppGpp help plants to function during nitrogen starvation by modulating photosynthetic performance and metabolite balance (Honoki et al. 2017).

### (p)ppGpp influence on chloroplast size and number

The molecular changes that occur in chloroplasts under (p)ppGpp accumulation affect chloroplast size and number. In an *Arabidopsis* line overexpressing *RSH3* (in WT), chloroplasts are significantly smaller and more numerous than in WT and display reduced chloroplast volume per cell volume. Similarly, in the *rsh1* mutant line, the chloroplast:cell volume ratio is decreased, and the opposite effect occurs for line overexpressing *RSH1*. This further corresponds with increased chlorophyll content in plants with mutations in genes coding for (p)ppGpp biosynthetic enzymes (i.e., *rsh2 rsh3*) but not in *rsh1* (Sugliani et al. 2016). *AtRSH3* overexpression in *rsh2 rsh3* background also results in reduced chloroplast size, however, with no apparent influence on chloroplast number per cell (Maekawa et al. 2015).

### Role of (p)ppGpp in chloroplast DNA replication

The inhibition of DNA replication is a hallmark of the stringent response in bacteria. In *B. subtilis,* direct binding of (p)ppGpp to DNA primase leads to inhibition of DNA replication elongation (Wang et al. 2007b). In *E. coli*, ppGpp impairs the DNA primase DnaG activity in vitro; however, in vivo only at the initiation stage. It was proposed that alarmones of the stringent response likely fail to inhibit the primase strongly enough, and therefore, to stop the elongation of DNA replication. In *E. coli*, (p)ppGpp might be mainly used for RNAP inhibition and therefore, likely, their amounts are not enough to inhibit DNA replication elongation (Maciag-Doroszynska et al. 2013). DNA replication is also regulated through transcription, likely via the (p)ppGpp-mediated RNAP inhibition, as in conditions promoting (p)ppGpp accumulation transcription of *E.coli dnaA* operon is diminished. DnaA is a replication initiation factor, which promotes the unwinding of DNA at *E. coli* origin of replication—*oriC*. Transcription form the *oriC* proximal *gid* and *mio*C promoters is also required for the replication of *oriC* plasmids and was shown to be inhibited by (p)ppGpp in vitro. That underscores again that in *E. coli* (p)ppGpp-mediated DNA replication is regulated at least at the initiation stage (Nazir and Harinarayanan 2016).

In *Arabidopsis,* the accumulation of (p)ppGpp—although it reduces chloroplast size and total volume per cell—increases their number without changing DNA content per plastid, suggesting increased chloroplast replication and division (Sugliani et al. 2016). Thus, despite the very high level of homology between bacteria and plants in the RSH domain structure and function as well as molecular targets of alarmones, (p)ppGpp-mediated responses in plants become different, according to Sugliani et al. (2016) likely due to the evolutionary gene transfer of vast amount of endosymbiont/plastid DNA into the host genome. Since chloroplasts do not proliferate as frequently as bacteria, it is also understandable that their DNA replication would be rather differently regulated. Sugliani et al. (2016) suggested that the inability of (p)ppGpp to inhibit DNA replication in chloroplasts may be caused by the lack of bacterial-like DNA primases in plants, which are essential components of replication machinery in bacteria and targets of (p)ppGpp. Another way to explain the lack of (p)ppGpp-mediated DNA replication inhibition in *Arabidopsis* plants is the expenditure of these nucleotides for the regulation of transcription or other molecular targets, similarly as it was proposed for *E. coli* (Maciag-Doroszynska et al. 2013).

## Role of alarmones in the expression of nuclear genes

The accumulation of (p)ppGpp in plants affects not only the transcription from chloroplast but also from nuclear genes. Surprisingly, among nuclear genes that transcription is upregulated in *Arabidopsis RSH3oe* (in WT) line, which accumulates (p)ppGpp and displays reduced chloroplast functions, are genes important for chloroplast functioning. Genes encoding proteins involved in transcription from chloroplast promoters, proteins important for mRNA, tRNA and rRNA processing, e.g., pentatricopeptide repeat proteins, which are involved in RNA processing, stability and translation in chloroplasts and mitochondria, are upregulated in the *AtRSH3oe* line. Also genes important for rRNA processing in cytosol are activated in that line. These expression studies suggest that the (p)ppGpp-invoked decrease in chloroplast transcription promotes nuclear compensatory mechanisms (Abdelkefi et al. 2017). This feedback effect requires retrograde signaling from the plastid to control nuclear gene expression. Since (p)ppGpp accumulation invokes multiple effects on metabolism and metabolic signatures were proposed, among other factors, to regulate retrograde signaling (Pfannschmidt 2010; Bobik and Burch-Smith 2015), (p)ppGpp may be important to trigger this pathway. Since intracellular signaling is necessary for coordinating cell responses to constantly changing environmental cues, (p)ppGpp production in chloroplasts may function to orchestrate plant adaptation and development.

The accumulation of alarmones in *Arabidopsis RSH3oe* (in WT) line leads also to a downregulation of vast number of nuclear genes. Among those are defense-related genes, such as LRR receptor kinases serving to recognize microorganism associated molecular patterns (flg22-induced receptor-like kinase 1—*FRK1*, chitin elicitor receptor kinase 1—*CERK1*, NSP interacting kinase 2—*NIK2*, suppressor of BIR1-1—*SOBIR1*). Moreover, transcripts of genes encoding for mitogen-activated protein kinase (MAPK) cascade proteins, important for pathogen-induced signal transduction, are downregulated. Similarly, transcription of genes involved in the biosynthesis of a plant defense hormone—SA (isochorismate synthase 1—*ICS1*, calmodulin binding protein 60g—*CBP60g*, SAR deficient 1—*SARD1*), SA signaling (*WRKY53*) as well as genes induced by the hormone (non-expressor of PR genes 1—*NPR1*, pathogenesis-related: *PR1*, *PR2*, *PR5*) is lowered. Nevertheless, among transcripts that expression decreases in the line accumulating alarmones are ones encoding for proteins involved in a negative regulation of defense responses and programmed cell death. The (p)ppGpp-invoked negative regulation of SA marker gene (*PR1*, *PR2*, *PR5*) expression is corroborated with their expression in *Arabidopsis RSH1*–*CRSH* quadruple mutant line, where it is significantly higher in comparison to WT plants. Similarly, PR1 protein level also tends to be higher in the quadruple mutant line than in the control (Abdelkefi et al. 2017).

Interestingly, transcripts of jasmonic acid (JA)-responsive genes are upregulated in the *RSH3oe Arabidopsis* line (Abdelkefi et al. 2017). Hence, on the first sight the experimental data appear contradictory, since it was mentioned in the earlier section [*(p)ppGpp regulate the level of hormones, lipids and metabolites in chloroplasts*] that (p)ppGpp accumulation leads to decrease in the level of 18:3 fatty acids (Maekawa et al. 2015). Therefore, it would also result in downregulation of the plastid product 12-oxo-phytodienoic acid, a JA precursor that is biosynthesized from linolenic acid (18:3), and further in the decrease of JA production in peroxisomes. Hence, JA-responsive genes should be rather downregulated in the *AtRSH3oe* line. However, the JA-responsive gene expression data was obtained with the *AtRSH3oe* line in the WT background where RSH3 is GFP-tagged (Sugliani et al. 2016), whereas the metabolite studies were performed with the *AtRSH3oe* line in the *rsh2 rsh3* background with native *RSH3* (Maekawa et al. 2015). Since both of these lines produce different levels of ppGpp (~ 7- and ~ 3-fold higher than WT plants, respectively) and exhibit to some extend different physiological phenotypes, it is hard to compare data from these two lines. Moreover, Sugliani et al. (2016) showed that in *Arabidopsis* plants containing a transgene encoding a chloroplast-targeted ppGpp synthetase from bacteria, under the control of a dexamethasone-inducible promoter, that exhibited phenotypes similar to the *AtRSH3oe* (in WT) line and produced very high amounts of ppGpp (~ 30-fold), *accD* transcripts are less abundant than in the line where the synthetase is catalytically inactive (Sugliani et al. 2016). Thus, it would be very interesting to check the expression of the discussed nuclear genes in the *AtRSH3oe* line in the *rsh2 rsh3* background.

Nevertheless, the expression data presented by Abdelkefi et al. (2017) confirms the existence of SA and JA signaling antagonism. Furthermore, it suggests that the over-accumulation of alarmones in *Arabidopsis* plants might regulate their resistance towards necrotrophs, since JA production in response to such pathogens promotes plants resistance to these microorganisms (Abdelkefi et al. 2017). It also implies that (p)ppGpp may regulate not only SA but also JA production in chloroplasts.

## Alarmones in cytoplasm

Interestingly, (p)ppGpp may also function in cytoplasm, promoting plant growth reduction. *Arabidopsis* transgenic plants that overexpress inducible *B. subtilis yjbM—*coding a (p)ppGpp synthetase that due to the lack of transit peptide is supposed to localize in cytoplasm—produce 10–20-fold higher levels of (p)ppGpp and display reduced fresh weight (about 20%) in comparison to WT plants. Hence, the authors proposed that depending on plastidial or cytosolic localization, (p)ppGpp either promotes or restrains plant growth, respectively, by regulating gene expression and metabolic processes to optimize growth in changing environmental conditions (Ihara and Masuda 2016). However, no localization studies of the YjbM protein in the *Arabidopsis* transgenic line were performed, leaving open the possibility of its plastidial functioning. Importantly, the proposed idea is based on comparison with the results obtained with *AtRSH3oe* line in the *rsh2 rsh3* background, which showed more robust growth than WT plants (Maekawa et al. 2015) and not with the *AtRSH3oe* line in WT background, whose growth was reduced in comparison to WT plants (Sugliani et al. 2016). Nevertheless, as it was already mentioned in the previous section, Maekawa et al. (2015) overexpressed native *RSH3*, whereas Sugliani et al. (2016) GFP-tagged one, suggesting that the GFP tag may influence localization of the RSH3 protein. However, *At*RSH3–GFP fusion protein transiently expressed in *Nicotiana benthamiana* localizes in chloroplasts (Maekawa et al. 2015). Nevertheless, since Sugliani et al. (2016) did not show the localization of *At*RSH3–GFP fluorescence, it leaves open the possibility of its cytosolic positioning and functioning to produce (p)ppGpp in cytosol, similarly to the *yjbM*-expression line (Ihara and Masuda 2016). It is also possible that the relatively lower increment of ppGpp (~ 3-fold higher than WT plants; Maekawa et al. 2015) promotes plant growth, whereas higher (~ 7-fold higher than WT plants; Sugliani et al. 2016) induces opposite effect.

Thus, it is possible that (p)ppGpp, or rather some upstream components that regulate (p)ppGpp localization, could serve as conductors that orchestrate plants decisions concerning energy investment in growth or survival, depending on (p)ppGpp presence in chloroplasts or cytoplasm, respectively. The idea of (p)ppGpp functioning in cytoplasm is supported with in silico analysis performed on the eukaryotic SAH MESH1, which showed that it does not carry the mitochondrial target peptide and thus suggested cytoplasmic localization of the protein (Atkinson et al. 2011). Similarly, the expression of *S. japonica RSH* in the yeast *Saccharomyces cerevisiae* results in (p)ppGpp accumulation solely in the cytoplasmic but not in the mitochondrial fraction (Ochi et al. 2012). Moreover, the deletion of *MESH1* in *Drosophila* induces retarded growth, resembling the phenotype observed in *Arabidopsis yjbM* transgenic line (Sun et al. 2010).

We can assume two ways of (p)ppGpp accumulation in cytoplasm. It can either be produced by RSH proteins during their cytoplasmic localization or transported to cytoplasm after chloroplastic synthesis, which is the case for other nucleotides (Ihara and Masuda 2016).

Both MESH1 and *At*RSH1 are (p)ppGpp hydrolases. No (p)ppGpp synthetase has been found in animals so far, which leaves us with a question on the function of MESH1 in the plausible non-(p)ppGpp environment. *At*RSH1 interacts with the cytoplasmic protein RPP5 involved in plant responses to pathogens, suggesting that it may function in cytoplasm. Thus, it is possible that (p)ppGpp hydrolases could function in cytoplasm to somehow cope with (p)ppGpp produced by bacteria during pathogenic infections, since (p)ppGpp production is important for bacterial virulence (Dalebroux et al. 2010). However, there is no available data that supports that hypothesis. Moreover, how would it happen that plant enzymes would act on inner bacterial components? Hence, analysis of (p)ppGpp accumulation on the site of the host and pathogen during infections are important to better understand the phenomenon of the stringent response.

Implicit is the idea that the stringent response in plants is not only a form of response to stress but also a way to regulate plant growth and development, likely through facilitation of the cross-talk between nucleus and chloroplasts. Similarly, in bacteria, basal levels of alarmones regulate cell growth and metabolism (Potrykus et al. 2011; Gaca et al. 2013). Furthermore, bacteria missing *RSH* are mostly obligate intracellular parasites or endosymbionts (Atkinson et al. 2011), implying the importance of RSH for the regulation of fundamental processes.

## Role of RSH and alarmones in plants

### Spatiotemporal expression of plant RSH

**Fig. 2:**
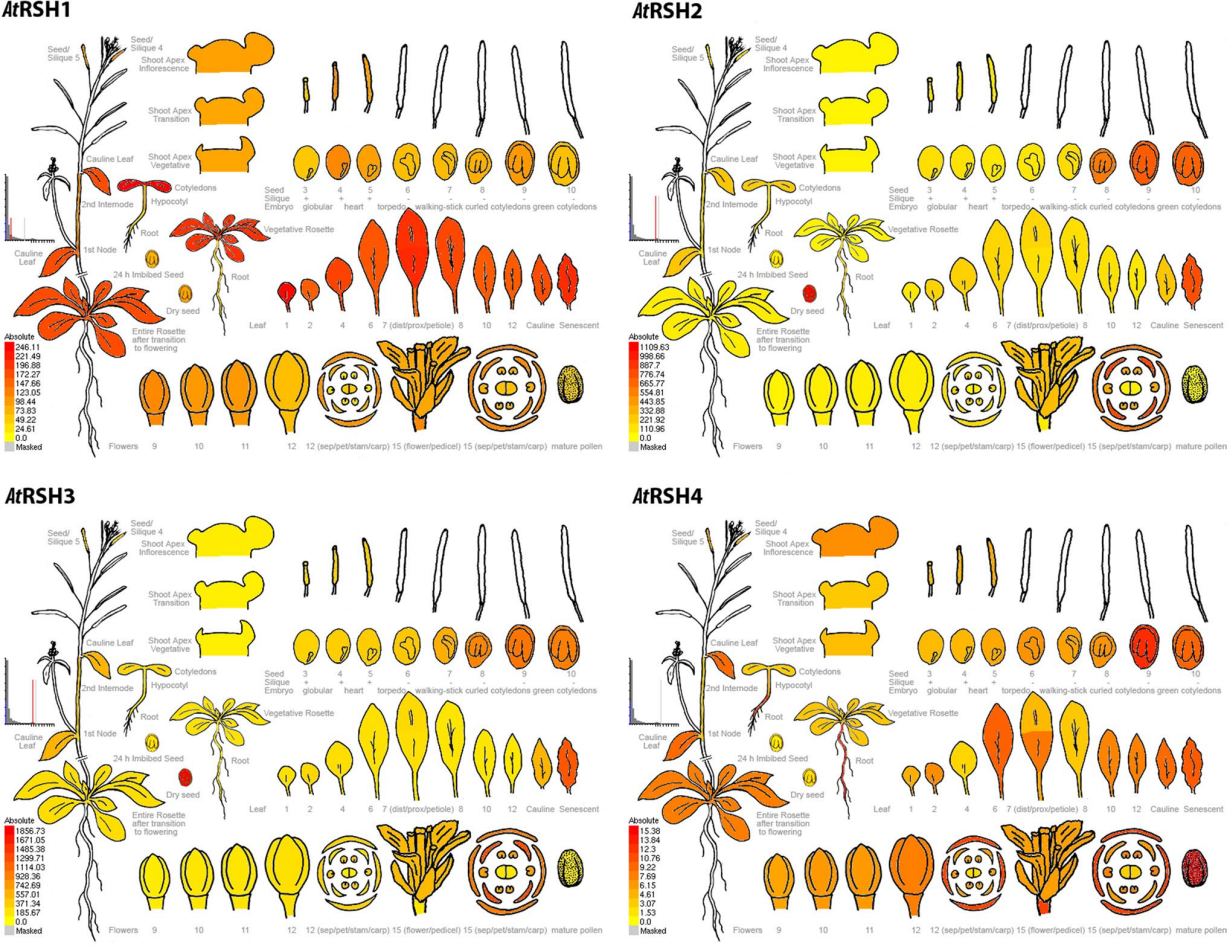
Electronic fluorescent pictographs (eFPs) for *Arabidopsis thaliana* RelA/SpoT homolog genes *RSH1* (*At*4g02260), *RSH2* (*At*3g14050), *RSH3* (*At*1g54130) and *RSH4* (*At*1g30850) transcript levels in different plant organs over various developmental stages. Expression levels between *RSH* are not normalized. High and no expression levels are indicated by red and yellow colors, respectively. For later stage siliques (6–10; corresponding to torpedo, walking-stick, curled and green cotyledons stages of embryo development) only the seeds were collected for analysis—not the siliques themselves. For stages 3–5 (corresponding to globular, heart and torpedo stages of embryo development) the seeds were collected with siliques. More detailed information about the microarray and other studies that are the sources for these developmental maps and further tissue-related information can be found at *Arabidopsis* eFP Browser at bar.utoronto.ca, in Gene Expression Map of *Arabidopsis* development (Schmid et al. 2005 and the Nambara Lab) and Winter et al. (2007)

An overview of developmental expression profiles for *Arabidopsis RSH* is available at the *Arabidopsis* eFP Browser (Schmid et al. 2005; Winter et al. 2007) at the Bio-Array source for Plant Biology (http://bar.utoronto.ca/welcome.htm) and presented here in Fig. 2. Developmental maps of *Arabidopsis RSH1*, *RSH2*, *RSH3* and *CRSH* demonstrate highly similar patterns of *AtRSH2* and *AtRSH3* expression, which reflects the results of in silico sequence and phylogenetic data analysis; this corroborates the suggestion that true *RSH3* is missing from the *Arabidopsis* lineage, and *AtRSH3* is actually the result of *RSH2* duplication (Atkinson et al. 2011). All four *RSH* are expressed at various stages of embryo development. *RSH1* expression fluctuates and reaches its peaks at heart and curled cotyledons stages and *CRSH* expression peaks in later stages of seed development (mostly curled and green cotyledons), whereas *RSH2* and *RSH3* display high expression at late embryo developmental stages (8–10; Fig. 2). While the high *RSH2* and *RSH3* expression continues in dry seeds, neither *RSH1* nor *CRSH* transcripts appear to be significantly conserved in the organ. Thus, the expression data suggests that RSH1 and CRSH might play a more important role in seed pre-dormancy, whereas RSH2 and RSH3 in seed dormancy or in the imbibing embryo. RSH2 and RSH3 could also possibly regulate plastid development during embryo maturation. *Arabidopsis* embryos display a transient green stage during seed formation. In that time chloroplasts are being formed, whereas in the late embryo development, during seed desiccation, they are de-differentiated into eoplasts that retain the transcriptional apparatus but disassemble the photosynthetic one. Since the primary chloroplast biogenesis in *Arabidopsis* embryos is not understood yet, it would be interesting to check whether (p)ppGpp could serve as the initiator/mediator of the chloroplast–eoplast transition. Since ABA is involved in seed maturation, dessication and dormancy and promotes *RSH2*/*3* expression (Yamburenko et al. 2015), it could regulate this process via the RSH2/3-(p)ppGpp module as it was proposed by Pfannschmidt et al. (2015).

*RSH1* expression during plant development is the most steady out of the four *Arabidopsis RSH*. Its highest levels are observed in cotyledons, first and second leaves, vegetative rosettes and leaves of plants after transition to flowering, including cauline leaves and ones undergoing senescence as well as in the first stem node and the second internode, and, to some extend in shoot apex, buds as well as flowers (mostly in sepals and petals) and pedicels. *CRSH* expression is quite distinct, since the highest number of *CRSH* transcripts is produced in roots and mature pollen, which according to eFP Browser data is not the case for other *Arabidopsis RSH*. Relatively high *CRSH* expression also occurs in particular rosette leaves after transition to flowering, including cauline and senescent leaves, as well as in inflorescent shoot apex, buds, flowers (mostly sepals) and pedicels. The highest *RSH2* and *RSH3* expression is reserved for the already mentioned later stages of embryo development, dry seeds and mature flowers (mostly petals, sepals and stamens). Similarly to *RSH1* and *CRSH*, very high *RSH2* and *RSH3* expression also takes place in leaves undergoing senescence (Fig. 2).

The expression levels of *RSH* in Fig. 2 are not normalized. Therefore, while comparing absolute expression values between all *AtRSH*, we observe that *RSH2* and *RSH3* transcripts are actually the most abundant *RSH* in *Arabidopsis* plants. Their expression in root, hypocotyl, cotyledon, cauline and senescing leaf, flower (sepals, petals, stamens), during seed development as well as in dry seed is higher than the expression of *RSH1* and *CRSH*. Additionally, we also notice higher *AtRSH1* and *AtRSH3* expression in the first and second leaves as well as in vegetative rosette in comparison to *AtRSH2* and *AtCRSH* (*Arabidopsis* eFP Browser).

The other experimental approaches confirm the array data on the expression of all *Arabidopsis RSH* in flowers and show additionally their expression in pistils. Furthermore, *RSH2* and *RSH3* expression was shown to fluctuate during flower development. Their expression was shown to be present in carpels and sepals of juvenile buds and later also in stamens of pollinated flowers. Moreover, their expression was observed also in developing pollen (Masuda et al. 2008a; Mizusawa et al. 2008). One of the reasons for *RSH* expression in flowers could be their importance for fruit development regulation. Most plastid genes (photosynthesis-related, transcription apparatus-coding, tRNAs) as well as RbcL protein in green tomato fruits are strongly downregulated in comparison to leaves (Kahlau and Bock 2008), suggesting that (p)ppGpp accumulation may trigger fruit development.

The combination of the array and other experimental data clearly demonstrates ubiquitous expression of plant *RSH* in green tissues. According to the latter ones, *Arabidopsis RSH1*–*RSH4* expression occurs in the early stages of seedling development (2–11 days) in the following pattern: *AtRSH1* and *AtRSH3* genes are highly expressed in hypocotyls, leaves, leaf veins and shoot apical meristems, whereas *AtRSH2* and *AtCRSH* are expressed in leaves and shoot apical meristems. However, *AtCRSH* expression declines 5 days after germination. Interestingly, *RSH* expression in shoot apical meristems is very pronounced, which is not effectively captured in the array data. In adult plants (50 days old), *AtRSH1*, *AtRSH2* and *AtRSH3*, but not *AtCRSH*, are strongly expressed in rosette leaves (Mizusawa et al. 2008). However, Western blot analysis performed with WT plants by use of anti-CRSH antibody also showed CRSH production in rosette leaves, next to its expression in cauline leaves, siliques, stems and flowers (Masuda et al. 2008a).

According to Mizusawa et al. (2008) only *AtRSH2* is expressed in roots of seedlings. The expression of *RSH2*, and to some extent *RSH3*, occurs in roots of mature *Arabidopsis* plants (Mizusawa et al. 2008), which remains in agreement with the array data showing their relatively highest expression in that organ in comparison to other *RSH* (*Arabidopsis* eFP Browser). Nevertheless, in other experimental approaches, *Arabidopsis RSH2* and *RSH3* and partly *RSH1* as well as *Oryza sativa CRSH1* transcripts were found in roots. Unluckily, in those studies, *AtCRSH* expression was not tested (Tozawa et al. 2007; Mizusawa et al. 2008; Chen et al. 2014). However, protein expression studies showed no detection of *At*CRSH in *Arabidopsis* roots (Masuda et al. 2008a). Thus, although *Arabidopsis CRSH* expression in roots is quite pronounced in the absolute array data, it does not appear so in vivo, which is likely due to its lowest relative expression in comparison to other *Arabidopsis RSH*. That, along with other *AtCRSH* expression data, implies that *AtCRSH* is a rather stress responsive gene.

### (p)ppGpp impact on plant growth and development

Chloroplastic phenotypes elicited by different (p)ppGpp levels have an impact on plant growth and development. The antagonistic role of *Arabidopsis* RSH1 vs. RSH2, RSH3 and CRSH in (p)ppGpp production is important for maintaining alarmone levels during vegetative tissue development. *Arabidopsis* plants overexpressing *RSH2* (*RSH2oe*) are pale and smaller in comparison to WT plants. However, the surface of *Arabidopsis rsh2 rsh3* or *rsh1*–*4* quadruple mutant plants is also significantly smaller than WT plants. Nevertheless, these mutants stay darker than *rsh1* or WT plants (Sugliani et al. 2016), underscoring that (p)ppGpp accumulation promotes the pale phenotype. *AtRSH3oe* (in WT) plants, similarly to *AtRSH2oe* (in WT), are pale and smaller than WT plants (Sugliani et al. 2016). RSH3oe plants in the *rsh2 rsh3* background have increased cell number and grow better than WT plants. Nevertheless, these plants also have lower chlorophyll levels and are pale with respect to WT plants, which again shows that (p)ppGpp accumulation invokes pale phenotype (Maekawa et al. 2015). The differences in RSH3 protein-overexpressing lines could be again explained with the fact that the first group overexpressed RSH2–GFP and RSH3–GFP, whereas the latter one native RSH2 and RSH3. Furthermore, as already mentioned, relatively lower increment of ppGpp (~threefold higher than WT plants; Maekawa et al. 2015) possibly promotes plant growth, whereas higher (~ 7-fold higher than WT plants; Sugliani et al. 2016) induces opposite effect. Maekawa et al. (2015) proposed that the increased plant size might be the effect of metabolite fitness, provoked by decreased photosynthesis. Plants of the *AtRSH3oe* (in *rsh2 rsh3* background) line grow also better in nitrogen starvation conditions, what is likely attributed to changes in photosynthetic activities and metabolite balance (Honoki et al. 2017). On the other hand, the results with the *AtRSH3oe* line (in WT) are supported with the studies on *P. patens* homologs of *AtRSH2*/*RSH3*, *PpRSH2a* and *PpRSH2b*, as their overexpressor lines show significant growth suppression in a glucose-concentration-dependent manner (Sato et al. 2015).

The impact of (p)ppGpp on plant growth and development may result from the regulation of hormone production, as fatty acids and nucleotides, the levels of which are regulated by (p)ppGpp, are precursors of plant hormones.

### Plant senescence, nutrient remobilization and relocation and seed development

*Arabidopsis RSH* have been shown to be involved in fertilization, seed development and plant senescence (Masuda et al. 2008b; Sugliani et al. 2016), what correlates with their expression during late plant development in flowers and leaves undergoing senescence (Schmid et al. 2005; Masuda et al. 2008b; Mizusawa et al. 2008) (Fig. 2).

*AtRSH1*–*4* mutant studies showed that RSH2, RSH3 and to some extent CRSH promote plant senescence, whereas RSH1 shows the characteristics of a negative regulator of this process, likely due to their ability to synthetize and hydrolyze (p)ppGpp, respectively. While *rsh1* knockout plants manifest accelerated senescence, *rsh2 rsh3* mutants as well as other *RSH1*–*RSH4* multiple *Arabidopsis* mutants carrying insertions in both *RSH2* and *RSH3* genes display delayed senescence and that effect is bolstered when *CRSH* is knocked down. Similarly, plants overexpressing *RSH1* do not senescence as fast as WT plants. Thus, we can call (p)ppGpp as positive regulators of plant senescence (Sugliani et al. 2016).

Increased levels of (p)ppGpp alone are not enough to trigger senescence, as plants that accumulate alarmones do not undergo senescence in vegetative tissues. The phenotype is rather obvious after flowering during the seed filling time. It is known that during senescence nutrients are remobilized from old tissues to reproductive ones (Lim et al. 2007). In *RSH* mutants with delayed senescence (*rsh2 rsh3*) or plants with the stay-green phenotype that die while being still green (*Arabidopsis* quadruple mutant *rsh1*–*4*), seed weight is significantly decreased compared to controls. This implies that (p)ppGpp is important for proper seed development and that seeds achieve their weight when alarmone accumulation stimulates nutrient remobilization from dying vegetative tissues into reproductive organs. Strong expression of *Arabidopsis RSH2* and *RSH3* during senescence, flower and seed development implies that (p)ppGpp may act as a “push” and/or a “pull” of nutrients from vegetative into reproductive tissues. However, the lines overexpressing *RSH2* and *RSH3* also produce significantly smaller seeds than WT plants, but that might be the result of overexpression per se (Sugliani et al. 2016).

The accelerated dark-induced senescence phenotype in *Arabidopsis rsh1* mutant and *RSH3* overexpressing plants, the two lines over accumulating (p)ppGpp, is coupled with accelerated Rubisco degradation, whereas in the line overexpressing *RSH1*, the Rubisco protein level increases along with darkness exposition time. This implies that (p)ppGpp during senescence promote not only nutrient reallocation but also remobilization by stimulating chlorophyll and Rubisco degradation (Sugliani et al. 2016). Therefore, the plant stringent response is not only linked to stress responses in plants but also required for optimal plant growth and development. Nevertheless, (p)ppGpp seem to act as signaling molecules that inform plants about the necessity of nutrient remobilization from vegetative into generative organs, which resembles their survival functions in bacteria.

Importance of (p)ppGpp production to exert plant senescence correlates with low levels of pyrimidine in senescing tissues (Greenberg 1997). As (p)ppGpp inhibit enzymes involved in purine biosynthesis, they may also affect levels of other nucleotides and thus accelerate plant senescence.

Since ABA induces both senescence and *AtRSH2* and *AtRSH3* gene expression, and gene expression profiles invoked by (p)ppGpp and ABA highly overlap (Maekawa et al. 2015; Yamburenko et al. 2015; Sugliani et al. 2016), we can assume that (p)ppGpp mediates ABA-induced senescence in plants.

*AtCRSH* was found to be important also for proper fertilization and silique formation as its knock down leads to abnormal flower development impeding pollination, significantly smaller than WT siliques and 300-fold reduction of seed quantity (Masuda et al. 2008a). However, lines retaining residual *AtCRSH* expression do not show that phenotype (Sugliani et al. 2016).

### Role of RSH proteins in plant stress response

Changes in *RSH* transcripts and proteins and in (p)ppGpp levels under various stimuli as well as plant *RSH* expression in *E. coli* or *S. cerevisiae* conferring tolerance to different cues suggest that plant RSH play a role in response to abiotic and biotic stress.

**Fig. 3:**
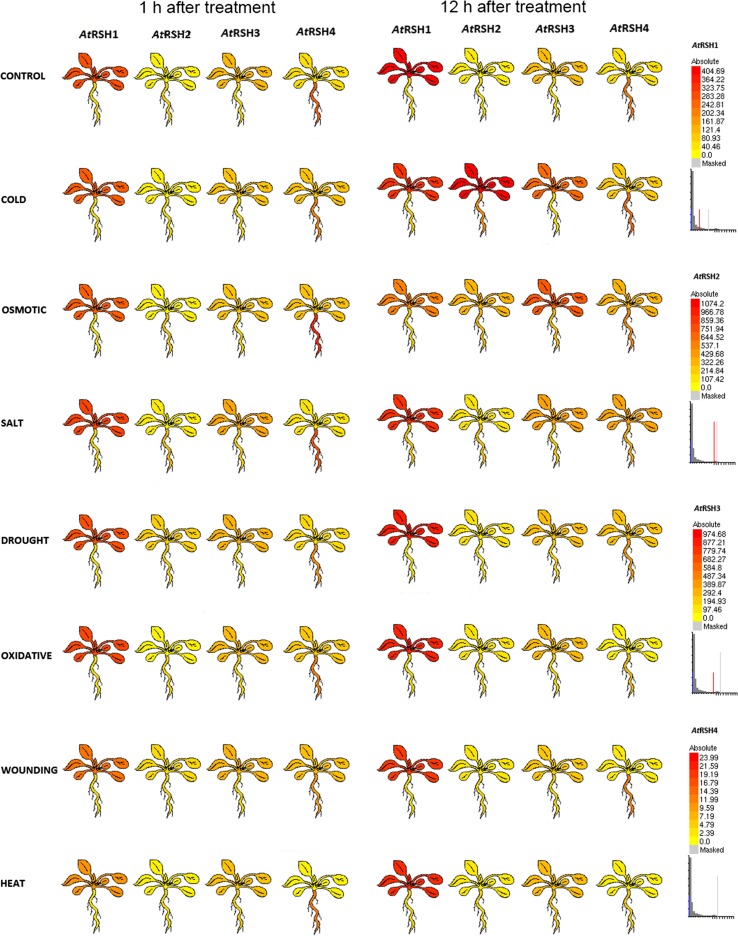
Electronic fluorescent pictographs (eFPs) for *Arabidopsis thaliana RSH1* (*At*4g02260), *RSH2* (*At*3g14050), *RSH3* (*At*1g54130) and *RSH4* (*At*1g30850) transcript levels under different stresses. 18 days after sowing and 3 h after dark/light transition *Arabidopsis* Col-0 plants treated with cold (plants transferred to ice for rapid cooling and kept at 4 °C in the cold room until harvest), 300 mM mannitol (osmotic stress), 150 mM NaCl (salt stress), drought (plants exposed to air stream for 15 min with loss of approximately 10% fresh weight), 10 μM methyl viologen (oxidative stress), wounding (leaves punctuation) or heat (38 °C for 3 h followed by recovery at 25 °C until harvest) conditions were harvested at 1 and 12 h post treatment. Expression levels between *RSH* are not normalized. High and no expression levels are indicated by red and yellow colors, respectively. More detailed information about the microarray and other studies that are the sources for these stress response maps and further information can be found at *Arabidopsis* eFP Browser at bar.utoronto.ca, in Winter et al. (2007) and Kilian et al. (2007)

In a yeast two-hybrid experiment, *A. thaliana* RSH1 protein was found to interact with RPP5, encoded by a member of disease resistance gene class, conferring resistance to pathogens such as *Peronospora parasitica*. However, no changes on the *AtRSH1* transcript level were observed under treatment with *Pseudomonas syringae* DC3000 or its derivative carrying *Avr*Rps4 as well as under factors known to exert some plant pathogen/insect-induced alike responses or mediate plant responses to pathogens, such as wounding or SA and methyl jasmonate, respectively (Gassmann et al. 1999; van der Biezen et al. 2000; Mizusawa et al. 2008). Moreover, the expression of *AtRSH1* was shown to even decrease under wounding and treatment with JA precursor 12-oxo-phytodienoic acid (OPDA) (Mizusawa et al. 2008; Chen et al. 2014) (Fig. 3). However, the data described here is based on *AtRSH1* mRNA expression studies, and RSH activity may also be regulated on the protein level, i.a., through protein interactions. Thus, the decreased *AtRSH1* transcript level in response to these stimuli does not exclude RSH1 protein from being active in these conditions. These stresses could stimulate RSH1 protein release from a complex or a cellular compartment, where it is kept inactive. Furthermore, the tested stress factors may induce RSH1 partners required for its activity. Although stress-induced cyanobacterium *Anabaena RSH* expression is not affected, marked increases in (p)ppGpp levels have been shown. The authors proposed that *Anabaena* RSH stimulates (p)ppGpp production in response to amino acid stress at the enzymatic but not the transcriptional level (Ning et al. 2011), which can explain the aforementioned *AtRSH1* expression. The *Anabaena RSH* expression vs. (p)ppGpp production can be also explained with the presence of another enzymes, which regulate (p)ppGpp metabolism. As an example, in *Thermus thermophilus*, a non-RSH enzyme HB8 a Nudix hydrolase was shown to regulate the level of ppGpp, and a homologous pyrophosphohydrolase, localized in chloroplast, was found in *Arabidopsis* to convert ppGpp into pGp, ppGp and pGpp (Ooga et al. 2009; Ito et al. 2012). Since a RelA/SpoT-independent ppGpp metabolic pathway exists in plants, it is plausible that (p)ppGpp level does not always correlate with *RSH* expression. However, considering that *At*RSH1 functions as (p)ppGpp hydrolase, it is understandable that its expression decreases under stress stimuli to promote alarmone production. Surprisingly, recent findings show that (p)ppGpp accumulation in *Arabidopsis* plants promotes their susceptibility towards TuMV, suggesting that (p)ppGpp may not always function as positive regulators of plant responses to stress. Nevertheless, upon the viral infection *AtRSH1* expression decreases, meaning there is no stimulation of (p)ppGpp hydrolysis. Why would plants promote the production of alarmones and create an environment hostile for pathogens is not clear. The environment rich in (p)ppGpp might be the result of TuMV action, which may hijack the plant effector-triggered immunity for the purpose of RSH1 degradation or retention in the cytoplasm. Decreased (p)ppGpp-invoked photosynthetic efficiency would further make the plant more vulnerable to the virus (Abdelkefi et al. 2017).

The expression of plant *RSH* from the RSH2/RSH3 subgroup is affected by SA and JA or its precursor. *Capsicum annum PepRSH*, a member of the subgroup, was found to be strongly induced 6–24 h after treatment with SA (Kim et al. 2009). However, as in the case of *AtRSH1*, *NtRSH2* expression did not change when tobacco plants were treated with the hormone (Givens et al. 2004). Similarly, treatment of *Arabidopsis* plants with methyl jasmonate had no impact on *AtRSH2* expression (Mizusawa et al. 2008), whereas it strongly affected the expression of *PepRSH* (Kim et al. 2009). Nevertheless, *AtRSH2* transcript level increases under treatment with OPDA, a JA precursor (0.5–1 h), and wounding (0.5–3 h), which is known to stimulate JA-mediated responses. Similarly, *PepRSH* expression increases after pepper plant wounding (Kim et al. 2009). However, wounding or OPDA treatment does not affect or even decrease, respectively, the expression of *AtRSH3* and *AtCRSH* (Mizusawa et al. 2008). The information on *Arabidopsis RSH2*, *RSH3* and *CRSH* expression under wounding correlates with the array data presented in Fig. 3; however, the increased *AtRSH2* expression presented in that figure is not as pronounced as in the above-mentioned experiments. Eight hours after treatment with JA, the expression of *NtRSH2* increases three–fourfold and lasts up to 48 h, which is also reflected in its protein level. The change in *NtRSH2* expression under JA suggests that it might be involved in response to pathogens, wounding, insects or UV light. Indeed, *N. tabacum* plants infected with *Erwinia carotovora* produce around tenfold higher levels of *Nt*RSH2 protein compared to non-infected plants (Givens et al. 2004). The plausible involvement of RSH2 proteins in JA-mediated plant responses to stress is supported with finding the *O. sativa AtRSH2* homolog in cDNA libraries prepared from rice plants treated with the hormone (Xiong et al. 2001). Oppositely, *P. patens RSH2a* and *RSH2b* showed no changes in expression under treatment with JA (Sato et al. 2015). Nevertheless, it does not exclude that the expression of these genes might be affected by OPDA treatment.

The expression of *RSH2* homologs under JA or its precursor treatment, along with the negative regulation of plastidial transcription and translation invoked by (p)ppGpp and JA as well as their involvement in plant senescence (Givens et al. 2004; Mizusawa et al. 2008; Zubo et al. 2011; Sugliani et al. 2016), suggest that JA-mediated responses involve RSH2-dependent (p)ppGpp production that further affects chloroplast transcription and translation and promotes plant senescence. Changes in chloroplasts accompanied with chlorophyll breakdown are also observed during programmed cell death and diseases. Knowing that (p)ppGpp accumulation promotes chlorophyll breakdown, it is tempting to speculate that alarmones could function in mediating apoptosis and disease (Greenberg 1997; Givens et al. 2004; Sugliani et al. 2016). That idea is corroborated with a decreased expression of the negative regulators of programmed cell death in the line over-accumulating (p)ppGpp (Abdelkefi et al. 2017).

*PepRSH* was also induced with elicitin extracted from *Phytophthora citrophthora*, a secretory protein from oomycetes that is known to trigger defense responses (Kim et al. 2009). Recent studies show that *Arabidopsis RSH* expression changes after plant treatment with TuMV. Under these conditions the level of *RSH2* transcript increases suggesting that the expression changes serve to produce (p)ppGpp. However, that expression pattern seems to be not in favor of plants, since (p)ppGpp accumulation increases plant susceptibility to TuMV (Abdelkefi et al. 2017).

Plant *RSH* were also tested for their involvement in responses to abiotic stimuli such as drought, salt, osmotic and oxidative stress as well as temperature change. According to the array data (Fig. 3), under drought, the expression of *Arabidopsis RSH1*, *RSH2* and *RSH3* does not noticeably change in comparison to their expression in non-treated plants. Interestingly, the expression of *CRSH* slightly decreases in roots 1 and 12 h post treatment (hpt). However, while it decreases in roots, it also slightly raises in shoots 12 hpt. In other studies, under drought treatment (dehydration on a paper towel), *Arabidopsis* plants were shown to produce higher amounts of *RSH2* and *RSH3*, with no major effect on the level of *RSH1* and *CRSH* transcripts (3–6 hpt) (Ito et al. 2012). However, in both experiments drought-inducing conditions varied.

In one study, treatment of *Arabidopsis* plants with 250 mM NaCl increased *AtRSH2* expression and had no impact on the expression of *AtRSH1*, *AtRSH3* and *AtCRSH* (Mizusawa et al. 2008), whereas in another study it significantly increased both *AtRSH2* and *AtRSH3* transcript levels but decreased *AtCRSH* and had no impact on *AtRSH1* expression (Ito et al. 2012). In subsequent study, treatment of *Arabidopsis* plants with 100 mM NaCl even reduced *AtRSH1* transcript levels (Chen et al. 2014). These data reflect the array data presented in Fig. 3, according to which *AtRSH1* expression is slightly reduced in *Arabidopsis* shoots 12 hpt with 150 mM NaCl; *AtRSH2* and *AtRSH3* transcripts are more abundant, majorly in roots and shoots, respectively, 1 and 12 hpt. The expression of *AtCRSH* resembles the peculiar situation of its expression under drought treatment. 12 hpt with NaCl *AtCRSH* transcript level increases in shoots in comparison to non-treated plants but decreases in roots (Fig. 3). *PepRSH* expression also increases after *C. annum* plants treatment with 200 mM NaCl, reaching its peak 12 hpt (Kim et al. 2009). *S.* *japonica RSH* (*SjRSH*), which protein exhibits high homology with *At*RSH2 and *At*RSH3, was found in a cDNA library obtained from plants grown in high salt conditions (450 mM NaCl). Furthermore, the expression of *SjRSH* in *E. coli* or *S. cerevisiae* conferred tolerance to salt and osmotic stress. Experiments conducted with *S. cerevisiae* expressing *SjRSH* showed that it promotes the expression of genes, whose products are known to be involved in yeast responses to stress, including high osmolarity glycerol-responsive mitogen activated protein kinase 1 (HOG1), the global regulator of salt stress-responsive genes. Furthermore, the expression of stress-responsive genes in the line carrying *SjRSH* increased even higher under salt stress conditions. That, along with the complementation studies showing the functionality of *Sj*RSH synthetase, suggests that *Sj*RSH-mediated (p)ppGpp production promotes the expression of stress responsive genes (Yamada et al. 2003). Interestingly, treatment of *N. tabacum* plants with 0.5% ethanol, which probably mimics osmotic stress, dehydration or hypoxia, promotes *NtRSH2* expression, both on the mRNA and protein levels (Givens et al. 2004). The involvement of *RSH* in responses to osmotic stress is also corroborated with their expression under 300 mM mannitol treatment that invokes osmotic stress (Fig. 3). Here, 12 hpt, while the expression of *AtRSH1* strongly decreases in shoots, *AtRSH2* increases both in shoots and roots, *AtRSH3* and *AtCRSH* increases in shoots. On the other hand, another two *RSH* coding for homologs from the RSH2/RSH3 group (i.e., *P. patens RSH2a* and *RSH2b*) showed constitutive expression under dehydration and no changes under high salt concentration (Sato et al. 2015).

*AtRSH2* was found to be highly upregulated under oxidative stress induced with paraquat (50 µM), which also caused a significant decrease in *AtCRSH* and *AtRSH1* transcripts (Ito et al. 2012). Similarly, *PepRSH* expression was also found as paraquat-induced (Kim et al. 2009). In the array study (Fig. 3), where the oxidative stress was induced with methyl viologen (10 µM), no major changes in transcript abundance were shown for *AtRSH1*, *AtRSH3* and surprisingly also for *AtRSH2*; decreased expression was found for *AtCRSH* in roots in comparison to non-treated plants.

Primarily, ABA (100 μM) was shown to promote the expression of *AtRSH2* and to not affect the levels of other *Arabidopsis RSH* transcripts (Mizusawa et al. 2008). However, recent studies have shown that the treatment of *Arabidopsis* plants with ABA (75 μM) leads to increased expression of both *RSH2* and *RSH3*, which depends on ABA signaling mediated by type 2C protein phosphatases PP2C (Yamburenko et al. 2015). Furthermore, all *P. patens RSH* clustered within RSH1–RSH4 subgroups are transiently induced under ABA treatment (100 μM) (Sato et al. 2015). Thus, RSH proteins might play a role in mediating ABA-regulated responses, such as the regulation of seed dormancy, germination, stomatal aperture as well as responses to stress, i.a., drought.

The expression of *Arabidopsis RSH* depends on temperature as cold, and to some extend heat treatment, affects their levels. In the array experiments, under heat, *AtRSH1* expression strongly decreases in shoots 1 hpt and that drop is also kept to some degree 12 hpt. *AtRSH2* and *AtRSH3* expression do not change in comparison to control plants, whereas *AtCRSH* decreases, especially in roots (Fig. 3). Similar results were shown with quantitative PCR experiments; however, no significant changes in *AtRSH1* expression were annotated, and surprisingly even a tendency for its increased expression (Ito et al. 2012). While the expression of *RSH* genes does not seem to be strongly regulated by heat under tested conditions, cold treatment upregulates the expression of *AtRSH2* and *AtRSH3* and to some extent *AtCRSH* (Fig. 3). However, cold treatment was found to have no impact on (p)ppGpp production in pea plants (Takahashi et al. 2004).

The involvement of plant (p)ppGpp in stress responses is supported with increased ppGpp levels in pea under various kinds of stresses, such as wounding (also confirmed for *A. thaliana*, spinach, tobacco, rice and wheat), heat shock, salinity, heavy metals, drought, UV light, treatment with JA (also in rice and wheat), ABA or ethylene. Interestingly, the natural auxin IAA acts rather oppositely to the above-mentioned hormones as it was shown to reduce ppGpp levels, probably due to its involvement in plant growth and development rather than stress response (Takahashi et al. 2004).

In pea chloroplasts, under JA treatment, (p)ppGpp production steadily increases, reaching its peak at 60 min (Takahashi et al. 2004). In bacteria, (p)ppGpp production in a nutrient-deficient state reaches its peak within 15 min (Ochi 1987). That shows the complexity of plant stringent response, resulting from nuclear and chloroplastic compartmentalization of its elements.

Alarmone levels are also light dependent (Takahashi et al. 2004; Ihara et al. 2015). Takahashi et al. (2004) showed that abrupt replacement of pea plants from prolonged light (12 h) to dark conditions increases ppGpp levels markedly, whereas prolonged darkness (12 h) causes their reduction. Ihara et al. (2015) detected large amount of ppGpp in *A. thaliana* during dark time periods (e.g., for 3–6 h in 12 h light/12 h dark conditions) rather than light time ones. Similarly, transient dark treatment at noon resulted in ppGpp accumulation. The diurnal rhythm-regulated ppGpp production resembles the expression of *A. thaliana RSH1*, *RSH2*, *RSH3* and *CRSH* genes that also changes during a day. The *AtRSH1* transcript level is the highest at dusk, whereas *AtRSH2*/*AtRSH3* at noon, and *AtCRSH* at midnight (Mizusawa et al. 2008; Chen et al. 2014). *At*CRSH is probably responsible for the production of alarmones at night, as its (p)ppGpp synthetic activity depends on calcium (Masuda et al. 2008a), whose concentration rises in chloroplast stroma 20–25 min after the transition from light to dark (Johnson et al. 1995). While *At*RSH2/*At*RSH3 are likely responsible for alarmone production during the daytime, *At*RSH1 expressed at dusk would then degrade them in the very early nighttime.

Expression data show that plant *RSH1*–*4* are differentially regulated under various stress conditions. While *RSH2* expression is induced under pathogen infection, wounding as well as JA and OPDA treatment, *RSH2* and *RSH3* are upregulated in response to drought, salt and osmotic stress, *RSH1* transcription is rather downregulated or not affected in these stresses, as the main function of the RSH1 protein is to degrade the stress-induced alarmones. *CRSH* expression under stress does not seem to be markedly changed in the time frames and the types of stress factors tested so far. However, interestingly, under salt or drought conditions, the expression of the gene decreases in roots, while it increases in shoots (Fig. 3). Thus, it is tempting to assume that *CRSH* transcripts are transported between those plant organs under stress.

**Fig. 4:**
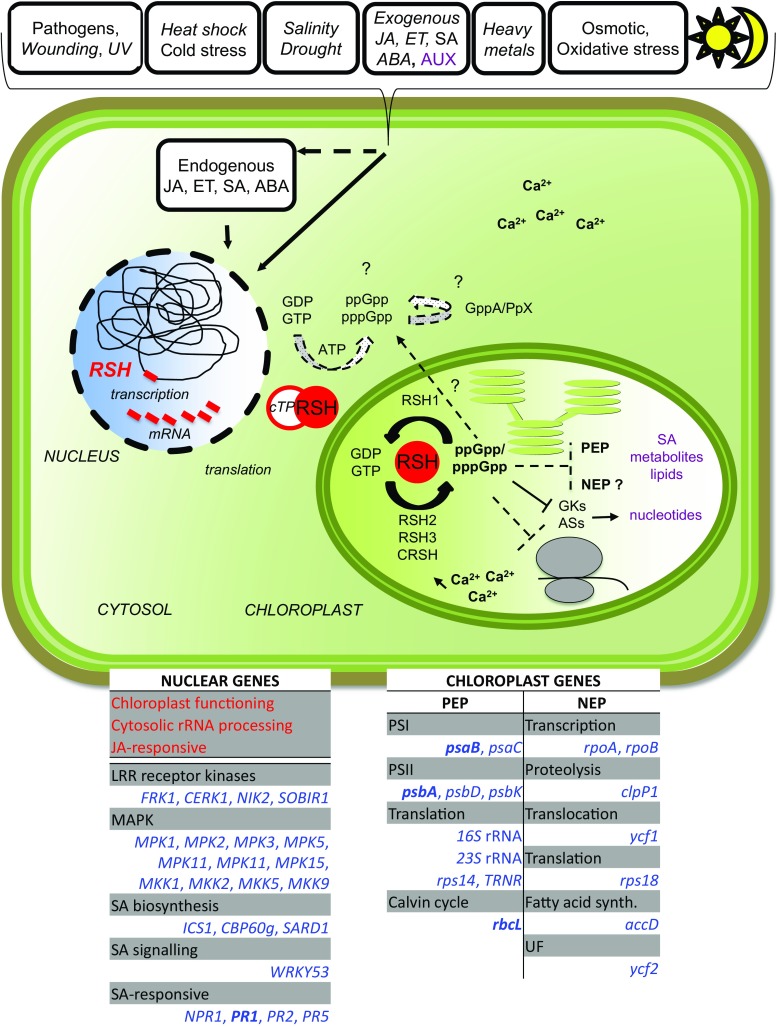
Overview of a proposed model for the plant stringent response. RSH1–CRSH are nuclear genes, whose expressions change during plant development and under stress. Based on *RSH* gene and protein expression studies as well as (p)ppGpp accumulation data under stress or hormone application, we propose that the stringent response in plants can be modulated by pathogens, wounding, UV irradiation, heat shock, cold stress, salinity, drought, exogenous hormone application (JA, jasmonic acid; ET, ethylene; SA, salicylic acid; ABA, abscisic acid: AUX, auxin), heavy metals, osmotic, oxidative stress and is additionally regulated by diurnal rhythms. Stress or hormone names written in italics were shown to increase (p)ppGpp concentration in plants. In that particular study (Takahashi et al. 2004), only the application of cold did not affect the level of alarmones, whereas SA, pathogens and osmotic and oxidative stress were not checked for the induction of (p)ppGpp production. Interestingly, exogenous auxin application blocks (p)ppGpp accumulation in pea plants (purple color). Since SA, JA and ET mediate pathogen-invoked responses, JA and ET wounding and heat shock-invoked responses, ET and ABA heat shock-prompted responses and ABA salt and drought-invoked events, these hormones were proposed to mediate the stress-induced plant stringent response, likely via regulation of the *RSH* gene expression. RSH proteins are translocated into chloroplasts, as they carry the chloroplastic transit peptide (cTP), where they regulate (p)ppGpp metabolism. RSH2/RSH3 along with CRSH function as the major (p)ppGpp synthetases and produce ppGpp and pppGpp from GDP and GTP, respectively, whereas RSH1 appears to be the main (p)ppGpp hydrolase. Possibly, (p)ppGpp also accumulate in cytoplasm either being produced there, before RSH translocation to chloroplasts, or in chloroplasts, from where they are transported to cytoplasm. Recently, plant genes encoding for possible pppGpp-specific phosphatases GppA/Ppx, which may function in cytoplasm to convert pppGpp to ppGpp, had been reported. In chloroplasts, (p)ppGpp affects transcription, downregulating the expression of PEP- and probably also NEP-dependent genes encoding for the elements of, e.g., PSI, PSII, translation machinery and others (table on the right bottom corner, blue color indicates decreased gene expression; functions of genes are described in gray fields). The regulation of transcription occurs either due to decrease in GTP level, a nucleotide important for the initiation of transcription from rRNA genes, for the sake of (p)ppGpp production, or via direct (p)ppGpp–PEP interaction. (p)ppGpp accumulation also influences nuclear gene expression (table on the left bottom corner, blue and red colors indicate decreased and increased gene expression, respectively). It promotes the expression of genes important for chloroplast functioning, cytosolic rRNA processing as well as JA-responsive. However, it leads to the reduction of transcripts of defense-related genes, such as *LRR* receptor kinases and *MPK*/*MKK* that serve to recognize microorganism associated molecular patterns and are important for signal transduction, respectively. It also downregulates the expression of genes encoding proteins involved in SA biosynthesis and signaling as well as responsive to the hormone. (p)ppGpp also regulate chloroplast translation, likely indirectly, as an effect of the negative regulation of transcription. However, (p)ppGpp may also inhibit translation directly, e.g., via binding to the elongation factor G. Chloroplast proteins found to be downregulated under (p)ppGpp accumulation are marked in the right bottom corner table in bold (PsaB, PsaA, RbcL). Additionally, the Rubisco small subunit (nucleus-encoded) and chloroplast f1 proteins (both NEP- and PEP-dependent), whose gene expression under (p)ppGpp accumulation was not shown so far, were assessed as downregulated under (p)ppGpp accumulation. Nuclear-encoded PR1 protein level was also shown as downregulated under (p)ppGpp over accumulation (marked in bold); however, it is likely the effect of decreased expression of the gene. (p)ppGpp inhibit GKs and ASs involved in GTP and ATP metabolism, respectively, and thus downregulate purine biosynthesis as well. Furthermore, (p)ppGpp accumulation in chloroplasts decreases SA level, the total lipids content as well as the levels of many other metabolites (purple color). In plants, many hormonal and environmental signals raise intercellular concentration of Ca^2+^, a messenger regulating cellular and developmental processes via Ca^2+^-binding proteins. One such protein is CRSH, whose (p)ppGpp synthetic activity depends on the calcium ion concentration. Since both cytosol and chloroplasts are loci of Ca^2+^ accumulation in plants, it is not clear whether CRSH is activated only via chloroplastic or both chloroplastic and cytosolic Ca^2+^ pools. PEP—plastid-encoded plastid RNA polymerase, NEP—nuclear-encoded plastid RNA polymerase, PSI—photosystem I, PSII—photosystem II, *rps14—*ribosomal protein 14, *TRNR—*arginine tRNA, *rbcL—*Rubisco large subunit, *rpoA—*RNA polymerase alpha subunit, *rpoB—*RNA polymerase beta subunit, *clpP1—*caseinolytic protease P1, *ycf1—*a subunit of the translocon on the inner envelope of chloroplasts, *rps18—*ribosomal protein 18, *accD—*acetyl-CoA carboxylase beta subunit, *ycf2—*ATPase of unknown function (UF), *FRK1—*Flg22-induced receptor-like kinase 1, *CERK1—*chitin elicitor receptor kinase 1, *NIK2—*NSP interacting kinase 2, *SOBIR1—*suppresor of BIR1-1, *MPK* (*MAPK*)—mitogen-activated protein kinase, *MPKK—*MPK kinase, *ICS1—*isochorismate synthase 1, *CBP60g—*calmodulin binding protein 60g, *SARD1—*SAR deficient 1, *NPR1—*non-expressor of *PR* genes 1, *PR1—*pathogenesis-related 1, *PR2—*pathogenesis-related 2, *PR5—*pathogenesis-related 5, AS—adenylosuccinate synthetase, GK—guanylate kinase

An overview of a proposed model for the plant stringent response and elements described here are depicted in Fig. 4.

## The role of alarmones in the virulence of plant pathogenic bacteria and plant resistance

(p)ppGpp-mediated stringent response is tightly linked with bacterial virulence and thus has an impact on plant vitality and/or survival (Dalebroux et al. 2010; Ancona et al. 2015; Chatnaparat et al. 2015). In plant pathogenic bacteria, (p)ppGpp is required for cell wall-degrading enzyme production, quorum sensing signal degradation, and Ti plasmid transfer (Zhang et al. 2004; Wang et al. 2007a). Bacterial (p)ppGpp production also plays a pivotal role in the expression of virulence genes, e.g., genes encoding for the type III secretion system (T3SS) (Ancona et al. 2015; Chatnaparat et al. 2015), which is a pathogenicity factor that enables Gram-negative bacteria the delivery of virulence proteins, called effector proteins, into plant cells to overcome plant defense responses that take place under the recognition of pathogen associated molecular patterns. The aim of the T3SS-mediated delivery of effector proteins into plant cells is to eventually acquire plant nutrients and cause disease (Bent and Mackey 2007; Monaghan and Zipfel 2012). In contrast, plants developed resistance proteins (e.g., RPP5) to recognize these effector proteins (e.g., *Avr*Rps4) and to trigger robust defense responses, including hypersensitive response. Hypersensitive response is a type of local, programmed cell death that occurs at the site of pathogen invasion, accompanied with defense responses in local and distant tissues (Mittler et al. 1997; Mur et al. 2008).

*Erwinia amylovora* bacterium WT strain that encounters the inhospitable environment of its host, e.g., pear fruit, produces (p)ppGpp. Recent studies demonstrated that ppGpp is required for full virulence of *E. amylovora*, since the expression of T3SS in *E. amylovora relA* ^−^ *spoT* ^−^ double mutant is highly downregulated in comparison to a WT strain on immature pear fruits. These bacteria were unable to multiply in a plant environment and to infect apple shoots or immature pear fruits as well as to induce hypersensitive response on tobacco leaves (Ancona et al. 2015). Similarly, in the *Pseudomonas syringae relA* ^−^ *spoT* ^−^ double mutant, which produces undetectable levels of (p)ppGpp, the expression of the T3SS encoding genes is strongly reduced in comparison to a WT strain, when these bacteria are infiltrated into bean leaves. Moreover, the cells of the mutant strain do not grow and are completely non-virulent when delivered into bean leaves and are not viable within 24 h after spray-inoculation onto bean and tobacco leaf surfaces (Chatnaparat et al. 2015). As for plant pathogenic bacteria, a plant surface is a stressful, nutrient-limiting environment, and the inability to multiply and colonize plant interior, due to the lack of (p)ppGpp-mediated T3SS expression, is likely the cause for the above-mentioned bacterial death.

The level of (p)ppGpp in bacteria populating plant surface increases, resulting in the reduction of bacterial amplification; on the other hand, it enables them to survive in difficult conditions and to colonize host tissues (Chatnaparat et al. 2015). Therefore, mutants that are not able to produce (p)ppGpp and to be concomitantly virulent could be used as live vaccines (Dalebroux et al. 2010). They could potentially serve as agents to prime plants for induced resistance. Another possibility to modify bacterial virulence in the RSH-(p)ppGpp module is to find components that are able to switch on RSH protein hydrolase activity, locking it in the synthetase switch off state, as shown for *Streptococcus dysgalactiae* subsp*. equisimilis* with an unusual (p)ppGpp derivative (Hogg et al. 2004).

Plants may probably regulate the level of microbial (p)ppGpp to overcome the assault of pathogens. They may either inhibit or promote pathogen (p)ppGpp production and thus reduce microbial virulence and survival on plants or slow down their growth, respectively. Plant secondary metabolites, namely, isothiocyanates (ITCs; e.g., sulforaphane, allyl isothiocyanate) promote (p)ppGpp production in the *E. coli* WT and human pathogenic enterohemorrhagic strain (EHEC). ITCs are produced by many plants, especially in the *Brassicale* order, by the hydrolysis of metabolites called glucosinolates and are generally known as antimicrobial and antiviral factors. ITCs inhibit *E. coli* growth likely via the sequestration of specific amino acids and thus exploitation of the (p)ppGpp-mediated stringent response (Nowicki et al. 2016). Sulforaphane inhibits a wide range of bacteria and fungi in vitro, likely via the above-mentioned mechanism, and protects *A. thaliana* plants against *Fusarium oxysporum,* since the glucosinolate biosynthesis mutant with decreased level of sulforaphane is more susceptible to the pathogen (Tierens et al. 2001). Furthermore, *Arabidopsis* tissues that undergo hypersensitive response release sulforaphane. It serves as an inducer of cell death in *Arabidopsis*, bean and sunflower leaves and thus protects plants against pathogen spread. It also primes *Arabidopsis* plant defense against a virulent isolate of oomycete *Hyaloperonospora arabidopsidis* (Andersson et al. 2015). In the future, it would be interesting to see whether plant-produced (p)ppGpp have a direct impact on pathogen virulence. Although it was shown that *E. coli* cells are impermeable for (p)ppGpp (Potrykus et al. 2011), it is still an open question. Recent studies show that the accumulation of alarmones in *Arabidopsis* plants increases their susceptibility towards TuMV. It implies that pathogens could have developed systems that make use of plant (p)ppGpp production to overcome plant immunity (Abdelkefi et al. 2017).

ITC-mediated (p)ppGpp production by EHEC bacteria also inhibits the entrance of a prophage, whose genome is integrated into the EHEC chromosome, into a lytic cycle. The prophage genome encodes for factors responsible for EHEC pathogenicity, which are active when the bacteriophage enters that lytic stage. Since (p)ppGpp inhibits the entrance into the lytic cycle, it simultaneously decreases EHEC pathogenicity (Nowicki et al. 2016). Thus, it seems that ITC-induced (p)ppGpp production may inhibit viral entrance into a cycle that enables spread and pathogenicity of viruses.

In light of the huge economical losses in agriculture caused by plant viruses, which account for the quarter of all known viruses and for 47% of plant emerging infectious diseases (Anderson et al. 2004; Gergerich and Dolja 2006), the search for factors that enable viral disease management is highly important. It would be interesting to know whether ITCs would be able to promote the intrinsic (p)ppGpp production in plants and whether it would protects them from pathogens. However, recent studies show that (p)ppGpp accumulation in *Arabidopsis* plants coincides with increased susceptibility to TuMV, whereas decreased levels of (p)ppGpp associate with increased resistance to the pathogen. It was proposed that “a reduced readiness” of the plant defense system of *Arabidopsis RSH3oe* plants makes them more susceptible to the virus (Abdelkefi et al. 2017). While *Arabidopsis RSH1*–*CRSH* quadruple mutant plants seem to be primed for increased resistance to the virus, RSH3oe plants are rather primed for increased susceptibility, since the levels of defense-related transcripts and the defense hormone SA are significantly lower in the *Arabidopsis RSH3oe* line in comparison to WT plants. Nevertheless, (p)ppGpp over-accumulating plants under the infection to some extend restore their defense capabilities. It was shown that the reduced expression of SA-induced pathogenesis-related genes in *AtRSH3oe* line observed in the non-treated plants changes under TuMV infection. These plants produce almost equal to WT plants amounts of *PR1*, *PR2* and *PR5* transcripts. Similarly, SA production under TuMV is not significantly changed in comparison to WT plants (Abdelkefi et al. 2017). It implies that the plant stringent response retains its original, bacterial function aiming to withstand or overcome stressful metabolic situations. Thus, in conditions of the very high (p)ppGpp levels plants would rather prefer to decrease the expression of defense-related genes for the sake of the ones involved in proper chloroplast functioning. Only after encountering pathogen attack, these plants would work towards restoration of defense capabilities to the level of WT plants. Importantly, the described here results were obtained with the *At*RSH3–GFP-tagged overproducing line (in WT; Sugliani et al. 2016), which as previously described exhibits different phenotypes than the line overproducing native RSH3 (in *rsh2 rsh3*) (Maekawa et al. 2015), likely due to relatively higher ppGpp levels. Thus, it would be very interesting to check these responses in the line overexpressing native *RSH3*. Furthermore, to fully understand the role of (p)ppGpp in plant immunity, studies with WT plants treated with various pathogens, followed by alarmone and gene expression level studies, are needed. The constant over-accumulation of alarmones in RSH3oe lines may not reflect the naturally occurring plant stringent response under pathogen attack.

(p)ppGpp is important not only for the establishment of virulence in pathogenic bacteria but also for the symbiosis between rhizobium and leguminous plants. In plant-associated pseudomonads and rhizobia, ppGpp affects epiphytic fitness, biocontrol activity, biofilm formation as well as nodulation (Moris et al. 2005; Vercruysse et al. 2011; Takeuchi et al. 2012).

## Future prospects

A milestone in the “area of the stringent response” would be the discovery and characterization of amino acid residues that are important for RSH CTD and N-terminal part intra-molecular interactions, plausible intermolecular interactions among individual RSH molecules and/or with other binding partners. Putative residues were already proposed based on in silico analysis (Atkinson et al. 2011); however, they need further characterization. Finding RSH interacting partners is crucial to fully elucidate RSH function, especially in stress responses.

An interesting model to study the domain structure and specialization of plant RSH proteins would be polyploid plants like *Brassica napus*, whose genome is a result of genome hybridization and chromosomal doubling followed by duplication (Chalhoub et al. 2014). It would be interesting to analyze RSH protein structural divergence and the expression of particular homologues and their role in response to different environmental cues in such a polyploid plant. The fact that EF-hand motif occurred among plants, whose genomes are known to undergo duplication, suggests that polyploid plants might have evolved even further towards RSH differentiation and specialization.

Another important area of research is the non-RSH-mediated (p)ppGpp metabolism. Candidate genes coding for proteins involved in the (p)ppGpp level regulation, acting most probably in cytoplasm as they do not contain the plastidial transit peptide, had been reported recently (Ito et al. 2017). The authors identified a possible pppGpp-specific phosphatase GppA/Ppx that has been conserved in plants (Fig. 4). However, to fully understand the plant stringent response, further analyses of these and other genes and their products involved in regulation of (p)ppGpp production are required.

Furthermore, the correlation analysis of bacteria and plant-produced (p)ppGpp during plant–microbe interactions would be essential to fully understand the evolutional interplay between plants and symbionts/pathogens. It is important to analyze whether plant-produced (p)ppGpp may directly influence bacterial virulence.

### *Author contribution statement*

GD conceived the idea of the review. JB prepared the initial outline. All authors provided feedback to improve the outline. JB composed the manuscript. JB and JP prepared figures. All authors read and approved the manuscript.

## Abbreviations

CTD: C-terminal domain
(p)ppGpp: Guanosine tetra- and pentaphosphates
RelA: *Escherichia coli* (p)ppGpp synthetase
RNAP: RNA polymerase
RSH: RelA/SpoT homolog
SAH: Small alarmone hydrolase
SpoT: *Escherichia coli* (p)ppGpp synthetase and hydrolase
TuMV: *Turnip mosaic virus*
WT: Wild type
